# Macrophage metabolism in inflammatory heart disease: new insights and therapeutic implications

**DOI:** 10.3389/fmed.2025.1664538

**Published:** 2025-10-08

**Authors:** Aocheng Xiang, Aie Chang, Jichang Zhou, Jin Jin, Xi Zhang, Qianyan Wang

**Affiliations:** ^1^Liyuan Cardiovascular Center, Liyuan Hospital, Tongji Medical College, Huazhong University of Science and Technology, Wuhan, China; ^2^Institute of Hematology, Union Hospital, Tongji Medical College, Huazhong University of Science and Technology, Wuhan, China; ^3^Department of Rehabilitation, Liyuan Hospital, Tongji Medical College, Huazhong University of Science and Technology, Wuhan, China; ^4^Department of Cardiovascular Surgery, Union Hospital, Tongji Medical College, Huazhong University of Science and Technology, Wuhan, China

**Keywords:** inflammatory, heart, disease, macrophage, metabolic, reprogramming, immunometabolism, therapeutic targeting

## Abstract

Macrophages are essential immune cells involved in pathogen clearance, initiation and resolution of inflammation, and tissue repair across multiple organ systems. They exhibit remarkable phenotypic diversity, encompassing classical M1 and M2 subtypes-further subdivided into M2a, M2b, M2c, and M2d-as well as newly identified subsets such as Mreg, M4, Mox, and Mhem, each with distinct functional roles. Emerging evidence highlights cellular metabolism as a central regulator of macrophage phenotype and function. Distinct metabolic programs underpin the polarization of M1 and M2 macrophages in response to environmental cues, thereby critically influencing disease progression and tissue outcomes. Cardiovascular disease remains a leading cause of morbidity and mortality worldwide. In the heart, macrophages represent a dominant immune cell population and play integral roles in both pathological injury and tissue regeneration. This review provides a comprehensive overview of macrophage ontogeny, phenotypic heterogeneity, and metabolic reprogramming, with a particular focus on their roles in inflammatory heart diseases. We synthesize current findings on how metabolic pathways shape macrophage behavior and function within the cardiac microenvironment and discuss the therapeutic potential of targeting macrophage metabolism to modulate inflammation, promote repair, and improve clinical outcomes in cardiovascular disease.

## 1 Introduction

Cardiovascular diseases remain a leading cause of morbidity and mortality worldwide, particularly in developed countries (1). Accumulating evidence indicates that immune cells-including macrophages, neutrophils, T Lymphocytes (T cells), and B Lymphocytes (B cells)-play critical roles in both the injury and repair phases of inflammatory heart diseases (2). Among these, macrophages represent the predominant immune cell population in the heart and are indispensable in shaping cardiac inflammation, remodeling, and regeneration (3–6). Notably, increasing attention has been directed toward the metabolic reprogramming of macrophages and its impact on cardiovascular pathophysiology (7–9). Macrophages are ubiquitously distributed throughout the body and serve as key mediators of innate immunity. Through phagocytosis, antigen presentation, and cytokine production, they orchestrate both proinflammatory and anti-inflammatory responses, while also facilitating tissue repair and homeostasis (10). Traditionally, macrophages have been broadly classified into two functional subsets: classically activated (M1) and alternatively activated (M2) macrophages (11). However, advances in immunology and transcriptomics have revealed a much more nuanced spectrum of macrophage phenotypes. M2 macrophages can be further subdivided into M2a, M2b, M2c, and M2d subtypes, each with distinct activation stimuli and functional roles. In addition, emerging subtypes such as regulatory macrophages (Mreg), M4, Mox, and Mhem have been identified, underscoring the complexity and plasticity of the macrophage population (12). Cellular metabolism is fundamental to maintaining survival, supporting biosynthetic demands, and driving immune cell function. In macrophages, metabolic pathways not only fulfill energy requirements but also shape immune phenotypes and functional responses. Recent studies have shown that macrophages undergo dynamic metabolic shifts in response to microenvironmental cues, and these shifts critically influence their role in disease progression and resolution (13).

In this review, we provide a comprehensive overview of macrophage ontogeny, phenotypic diversity, and functional specialization, with a particular focus on the metabolic programming that underlies these states. We further examine how macrophage metabolism contributes to the pathogenesis and resolution of inflammatory heart diseases, highlighting potential therapeutic targets to enhance cardiac repair and improve clinical outcomes.

## 2 Metabolism directs the function and polarization of macrophages

### 2.1 The functions of macrophages

#### 2.1.1 Sources of macrophages

Macrophages are crucial components of the innate immune system. They can be classified into two categories: those derived from monocytes and those derived from tissue-resident macrophages (TRMs).

Generally, monocytes originate from the bone marrow. In the bone marrow, monocyte development includes the following progenitor cells: common myeloid progenitors (CMPs), granulocyte–macrophage progenitors (GMPs), and monocyte–macrophage and dendritic cell (DC) precursors (MDPs) (14). MDPs can differentiate into common monocyte progenitors (cMoPs), which may give rise to monocyte precursors. These precursors then develop into Ly6C^hi^ monocytes, migrate from the bone marrow and circulate in the blood, and after a few days, they enter tissues and differentiate into macrophages (15). Monocyte-derived macrophages play a key role in clearing pathogens and cellular debris, antigen presentation, and initiating adaptive immune responses (16).

TRMs typically enter organs during embryonic or early postnatal development and exist largely independently of monocyte input throughout life (17). Many TRM populations (in the heart, lungs, liver, kidneys, and brain) originate from fetal liver or yolk sac progenitors (15, 18). There are also populations of TRMs (in the stomach, intestine, and colon) that originate from peripheral monocytes and are subsequently maintained through local proliferation (19, 20). TRMs are often replenished by monocytes, especially in infection or injury. During inflammatory disease, the initiation of inflammation in different tissues leads to a rapid decline in TRMs (21). At this time, inflammatory monocytes/macrophages are recruited to the site of inflammation, where they play an important role in immune defense but may also contribute to the pathogenesis of various inflammatory diseases and organ failure (22). In acute lung injury, peripheral blood monocytes can differentiate into interstitial macrophages and alveolar macrophages, and interstitial macrophages can further differentiate into alveolar macrophages (23). TRMs acquire unique functions on the basis of the organs in which they reside (24). In basal states, TRMs are considered functionally immunosuppressive; they play important roles in maintaining homeostasis, inhibiting T cell activation, and promoting the resolution of inflammation (25). However, in inflammatory or pathological conditions, the functions of TRMs can be altered significantly. For example, in the tumor microenvironment, TRMs can be reprogrammed to exhibit pro-tumorigenic effects by promoting angiogenesis and tumor invasion (26, 27). During inflammation, Alveolar macrophages have also been shown to initiate inflammation by secreting C-X-C Motif Chemokine Ligand 1 (CXCL1), which enhances the recruitment of neutrophils (21).

#### 2.1.2 Polymorphism of macrophages

Monocyte-derived macrophages differentiate into different phenotypes on the basis of various environmental stimuli, with the most classic classification being M1 and M2 macrophages (28). In addition to the classic classification, Mreg macrophages are another newly identified type (29) (Table 1).

**Table 1:** Overview of the macrophage phenotypes and functions.

**Phenotype**		**Stimuli**	**Cell expression markers**	**Cytokines, chemokines, and other secreted mediators**	**Functions**	**References**
M1		PAMPs, DAMPs, IFN-γ, TNF, LPS	CD80, CD68, MHCII, iNOS	IL-1β, IL-6, IL-12, IL-23, TNF-α, Ro, RNS	Promotes inflammation, induce Th1 cell polarization	(30–36)
M2	M2a	IL-4, IL-13	CD206, CD209, Dectin-1	TFG-β, IL-10, CCL17, CCL18, CCL 22, Arg 1	Tissue repair and antifungal responses	(12, 28, 35, 44)
	M2b	IL-1R, TLR agonist	LIGHT, SPHK1, CD86, MCHII	TNF-α, IL-1b, IL-6, IL-10	Immunomodulation	(12, 28, 40, 44)
	M2c	IL-10, Glucocorticoids	CD163, MerTK, TLR1, TLR8	IL-10, TGF-β, CCL 16, CCL 18, CCL 13	Anti-inflammatory and phagocytic effects	(28, 44)
	M2d	A2R, IL-6	CD163, CD14	IL-10, TGF-β, VEGF, CCL18	Angiogenesis, tumor progression	(28, 35, 44)
Mreg		LPS, IFN-γ, immune complex	CD80, MHC-II, DHRS9	IL-10, TGF-β, NOS, IDO	Immunomodulation, antigen presentation	(28, 35, 46, 47, 332)
TRMs		According to specific phenotype	According to specific phenotype	According to specific phenotype	According to specific phenotype	(37, 47, 333)
M4		CXCL-4	CD68, calcium-binding protein S100A8, MMP7	IL-6, TNF, MMP7	Promotes inflammation, pro-atherogenic	(12, 52, 334)
Mox		oxPL	MR, CD163, HMOX1, Srxn1, Txnrd1	Mox, COX-2	Lower chemotactic and phagocytic capacity	(53, 334)
Mhem		Heme	CD163, HMOX1	IL-10, HMOX1	Anti-inflammatory, prevent foam cell formation	(54, 56, 57, 334)
M(Hb)		Hb/Hp	MR, CD163	IL-10, IL-1Ra, VEGF, IL-1β, lower ROS		(12, 54, 55, 334)

M1 macrophages arise in response to activation by pathogen-associated molecular patterns (PAMPs), damage-associated molecular patterns (DAMPs), and proinflammatory cytokines (such as interferon-γ, IFN-γ and tumor necrosis factor, TNF) (30). Following LPS activation, Fcγ receptor-mediated phagocytosis and Mitogen-Activated Protein Kinase (MAPK), Janus Kinase 1(JAK1), and JAK3 signaling are activated in M1 macrophages (31). These pathways control several inflammatory genes, endowing macrophages with proinflammatory characteristics (32, 33). M1 macrophages play crucial roles in defending against pathogens and initiating inflammatory responses. These cells express markers such as Cluster of Differentiation 86 (CD86), CD68, MHC class II molecules, and inducible nitric oxide synthase (iNOS). Moreover, they secrete high levels of proinflammatory cytokines, including interleukin-1β (IL-1β), IL-6, IL-12, IL-23, and TNF-α, thereby promoting inflammation and cytotoxicity (34). M1 macrophages can produce abundant reactive oxygen species (ROS) and reactive nitrogen species (RNS) to combat pathogen invasion. Concurrently, they secrete proinflammatory cytokines and stimulate the polarization of T helper 1 (Th1) cells, resulting in robust inflammation (35). However, the excessive proinflammatory and cytotoxic actions of these macrophages can lead to severe tissue damage (36).

In contrast to M1 macrophages, M2 macrophages play a central role in tissue repair, immune regulation, and fibrosis (37). These cells are typically activated by cytokines secreted by T helper 2 (Th2) cells and exhibit reduced antigen-presenting capabilities along with elevated levels of anti-inflammatory cytokines, such as IL-10 and transforming growth factor-β (TGF-β) (38). This anti-inflammatory effect helps to balance excessive inflammatory responses. However, the overactivation of M2-like macrophages can lead to tissue fibrosis, potentially resulting in organ dysfunction (35). M2 macrophages can be divided into four subtypes, M2a, M2b, M2c, and M2d macrophages, each with distinct functions (28, 39). M2a macrophages are induced by IL-4 and IL-13, M2b macrophages are activated by IL-1R or TLR agonists, M2c macrophages are induced by IL-10 and glucocorticoids, and M2d macrophages are induced by A2 adenosine receptors (A2Rs) and IL-6 (28).

M2a macrophages express high levels of the mannose receptor (CD206) and secrete profibrotic factors, such as TGF-β, which promote tissue repair (12). M2a macrophages can activate the STAT6 pathway through the coreceptor IL-4Rα, a pathway that primarily mediates tissue repair and antifungal responses, inducing the production of arginase 1 (Arg 1). Following this, arginine is degraded into polyamines and proline. These byproducts increase cell division and the accumulation of collagen fibers, which are vital processes for the repair and regeneration of tissues (35).

M2b macrophages are phenotypically and functionally similar to Mregs and are capable of producing both proinflammatory and anti-inflammatory cytokines, such as TNF-α, IL-1β, IL-6, and IL-10 (12, 40). In lupus nephritis, Granulin (GRN) enhances M2b polarization, leading to increased release of pro-inflammatory factors, which in turn worsens kidney inflammation and fibrosis (41). However, in the myocardial ischemia-reperfusion injury model, α-lipoic acid (ALA) induces M2b polarization, reducing inflammatory damage to cardiomyocytes and promoting IL-10-mediated immune tolerance. This will improve heart function (42).

M2c macrophages, also known as deactivated macrophages, possess anti-inflammatory functions. They secrete IL-10 and TGF-β and effectively phagocytose and eliminate apoptotic cells (28). Recent studies reveal that peroxisome proliferator-activated receptor γ (PPARγ)-dependent lipid synthesis is crucial for their repair functions—dephosphorylation of PPARγ T166 enhances fatty acid metabolism, fueling the production of PDGF, TGF-β, and VEGF to support tissue regeneration in skin and liver injuries (43).

M2d macrophages exhibit phenotypic and functional resemblance to tumor-associated macrophages (TAMs) (35). They secrete IL-10, TGF-β, and vascular endothelial growth factor (VEGF), promoting angiogenesis and tumor metastasis (44). For example, in gastric cancer, M2d macrophages can secrete these cytokines to promote tumor proliferation and metastasis (44, 45).

Mregs, a special subtype, are primarily formed through the differentiation of bone marrow precursors and peripheral blood mononuclear cells in *in vivo* microenvironments under special circumstances (35). Dehydrogenase/Reductase Member 9 (DHRS9) is a unique and stable marker of Mregs (46). Mregs possess distinct immunosuppressive functions (47) and play a key role in immune responses, especially during the process of tissue damage repair. A notable characteristic of Mregs is their high expression of MHC-II and CD80 molecules, which not only endows them with potent immunosuppressive capabilities but also enables them to effectively present antigens (46). Mregs can suppress immune responses both directly and indirectly. They can directly inhibit the proliferation of activated allogeneic and xenogeneic T lymphocytes and can also indirectly suppress immune responses by inducing regulatory T cells (Tregs) or by secreting molecules such as IL-10, TGF-β, NOS, and Indoleamine 2,3-Dioxygenase (IDO) (28, 35, 47).

Unlike monocyte-derived macrophages, TRMs present distinct phenotypes under homeostatic conditions. TRMs are characterized primarily by the expression of different markers according to specific phenotypes; monocyte-derived macrophages are characterized by the expression of CD11b, CD209, CD64, and c-mer tyrosine kinase (MerTK) (47). TRMs are divided into different subpopulations, such as Kupffer cells in the liver, alveolar macrophages in the lungs, microglia in the brain, and macrophages in the heart, on the basis of their anatomical location and function (37). TRM is a complex cell population. The current definition of TRM is not complete, and markers vary across different organs. For example, kupffer cells in the liver highly express inhibitor of DNA binding 3 (ID3) and Dectin1; alveolar macrophages express CD11c, sialic acid-binding Ig-like lectin F (Siglec-F) (in mice) or CD169 (in humans); and microglia express purinergic receptor P2Y, G-protein coupled, 12 (P2RY12) and transmembrane protein 119 (TMEM119) (48–50). Therefore, further researches are needed to provide accurate clues. Studies show that TRMs can also polarize according to their functions. For instance, alveolar macrophages can be categorized into two groups: M1 macrophages, which are characterized by pro-inflammatory and profibrotic activities and actively recruit neutrophils, and M2 macrophages, which exhibit anti-inflammatory and anti-fibrotic properties, promote asthma development, and facilitate tissue regeneration and resolution of inflammation (21). Microglia in the M1 polarization state exhibit strong pro-inflammatory and phagocytic functions, whereas in the M2 polarization state, they possess anti-inflammatory and tissue repair functions (51).

In human and murine atherosclerotic lesions, other types of macrophages have been identified, such as M4, Mox, Mhem, and M(Hb) macrophages (12). M4 macrophages, which are induced by the platelet chemokine CXCL-4, primarily express CD68, the calcium-binding protein S100A8, and matrix metalloproteinase (MMP) 7 in the adventitia and intima of arteries, and they are associated mainly with plaque instability (52). Mox macrophages have been identified in the atherosclerotic plaques of mice, where they are induced by oxidized phospholipids (oxPL). Mox macrophages account for up to 30 % of total plaque macrophages, and their specific functions remain to be further investigated (53). M(Hb) and Mhem macrophages are two subtypes of macrophages found in atherosclerotic plaques that coexist at sites of neovascular bleeding or unstable plaques (54). M(Hb) macrophages, which are stimulated by hemoglobin, typically express high levels of mannose and CD163 receptors and are involved in the clearance of hemoglobin/haptoglobin (Hb/Hp) complexes after plaque hemorrhage (55). After Hb/Hp complexes and erythrocytes are engulfed, the released heme can shift macrophages toward the Mhem phenotype (56). This particular subtype is capable of inhibiting the development of foam cells and offers specific protective benefits against atherosclerosis (57).

### 2.2 Metabolism directs the function and polarization of macrophages

**Figure 1:**
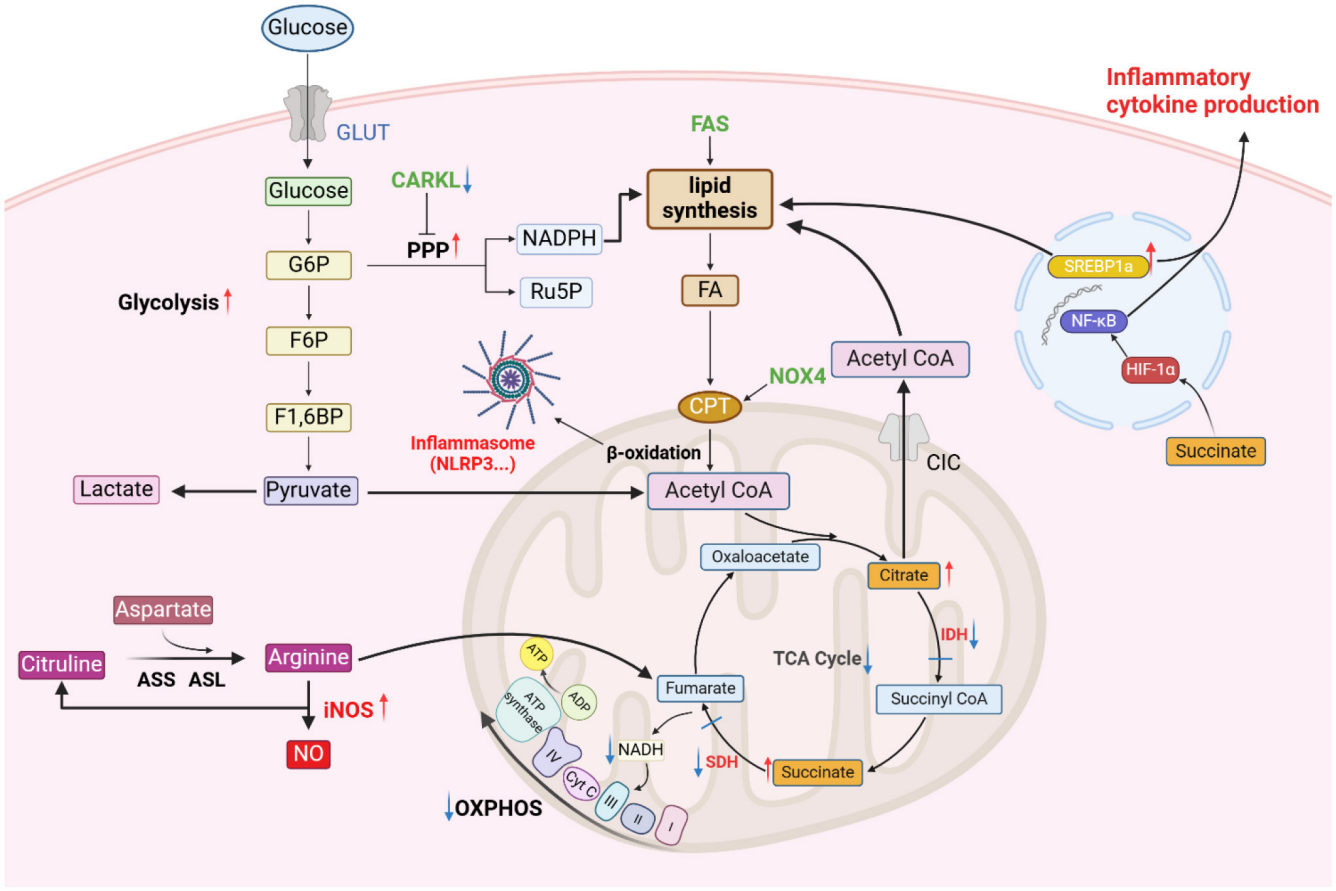
Metabolism in M1 macrophages. M1 macrophages exhibit increased glycolysis; an increase in CARKL leads to increased flux through the PPP. Owing to the suppression of IDH and SDH production, the TCA cycle is interrupted, leading to the accumulation of citrate and succinate. Citrate is exported from the mitochondria via a citrate transporter and participates in lipid synthesis. The accumulation of succinate stabilizes HIF-1α, inducing the activation of NF-κB, which promotes inflammation. The increased expression of SREBP-1α leads to increased fatty acid synthesis. FAO is also increased, facilitating inflammasome activation. M1 macrophages highly express iNOS, promoting the synthesis of NO from arginine, which further enhances inflammation (figure was created with Biorender.com).

**Figure 2:**
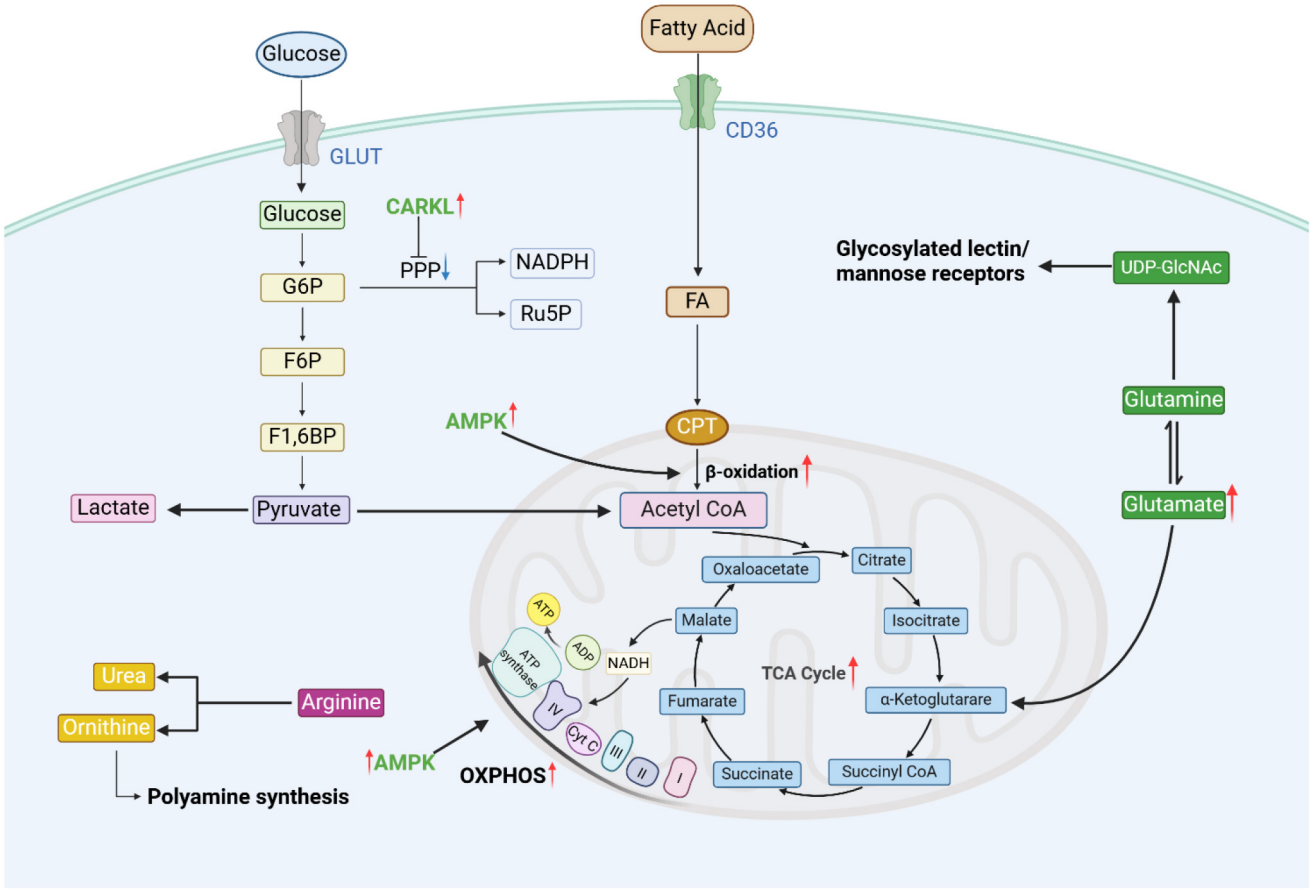
Metabolism in M2 macrophages. M2 macrophages exhibit reduced expression of CARKL, leading to the inhibition of the PPP. They have an intact TCA cycle. AMPK is involved in the activation of OXPHOS and FAO, which are their primary functional pathways. They also highly express arginase, which catalyzes the hydrolysis of arginine. Glutamine metabolism is increased, promoting the epigenetic activation of M2-type genes in macrophages (figure was created with Biorender.com).

Traditional cellular metabolism includes anabolism and catabolism, which produce ATP and biosynthetic precursors necessary for cell maintenance and growth. Like those of most cells, the metabolic pathways of resting macrophages are primarily based on the uptake of glucose and fatty acids. The aerobic oxidation of glucose and β-oxidation of fatty acids, accompanied by a robust tricarboxylic acid (TCA) cycle and mitochondrial oxidative phosphorylation (OXPHOS), continuously generate ATP to supply energy. An increasing number of studies indicate that metabolic processes and reactions play broader roles in immune effects (58). With the alteration of homeostasis in the body and the infiltration of related pathological products and exogenous substances, macrophages can activate their associated metabolic patterns to adapt to these changes (59). M1 and M2 macrophages have distinct metabolic characteristics depending on their degree of polarization. Typically, M1 macrophages express iNOS to produce nitric oxide (NO) from arginine. They exhibit enhanced glycolytic metabolism, an active pentose phosphate pathway (PPP), fatty acid synthesis, and impaired function of the TCA cycle and OXPHOS (60) (Figure 1). In contrast, M2 macrophages metabolize arginine through Arg-1, which is characterized by increased OXPHOS, fatty acid synthesis, and glutamine metabolism, as well as a suppressed PPP (60) (Figure 2).

#### 2.2.1 Characteristics and metabolism of macrophages

##### 2.2.1.1 Glucose metabolism

Various types of macrophages harness distinct glycolytic pathways to derive energy. M1 macrophages exhibit increased glycolytic activity, which is associated with their proinflammatory functions (60). This reliance on glycolysis, despite its lower ATP yield compared to oxidative phosphorylation, may prioritize speed over efficiency—a logical adaptation for rapidly responding to pathogens or tissue damage. LPS-induced bone marrow-derived macrophages (BMDMs) exhibit increased glycolytic metabolism (61). In M1 macrophages, the production of isocitrate dehydrogenase (IDH) and succinate dehydrogenase (SDH) is inhibited, leading to the disruption of the TCA cycle and the accumulation of citrate and succinate (59). The accumulated citrate is exported from the mitochondria via a citrate transporter involved in fatty acid production (62). This citrate shunt likely supports membrane biogenesis and prostaglandin synthesis, underscoring how metabolic intermediates directly influence inflammatory output. Activated M1 macrophages increase succinate levels, which stabilize hypoxia-inducible factor 1-alpha (HIF-1α), further promoting glycolysis (63, 64). HIF-1α is a transcription factor essential for the induction of multiple enzymes involved in glycolysis and may also be involved in the activation of NF-κB (63). High glucose-6-phosphate dehydrogenase (a key enzyme in the PPP) expression also activates the NF-κB signaling pathway, leading to intracellular oxidative stress and the induction of M1 macrophage polarization (62, 65). In contrast, M2 macrophages have an intact TCA cycle and can obtain the required energy through oxidative phosphorylation (66). The metabolic dichotomy between M1 and M2 macrophages mirrors their functional divergence: M1 cells sacrifice efficiency for rapid effector functions, while M2 cells prioritize energy conservation for tissue repair and homeostasis. M1 macrophages can produce nicotinamide adenine dinucleotide phosphate hydrogen (NADPH) through the PPP, which is involved in fatty acid synthesis for the production of inflammatory mediators and cell membrane remodeling (67). LPS-stimulated BMDMs exhibit increased PPP flux (68, 69). Carbohydrate kinase-like protein (CARKL), a protein resembling a carbohydrate kinase, restricts the PPP. Haschemi et al. found that CARKL expression decreases significantly during LPS-induced macrophage activation, triggering pro-inflammatory factor production. Conversely, when BMDMs are activated by IL-4, CARKL expression rises, helping to suppress the PPP (68). Studies have also found that macrophages induced by OxPL, known as Mox macrophages, exhibit unique metabolic features that are distinct from those of the traditional M1 and M2 macrophages. Mox macrophages rely on glucose metabolism and the PPP to support the production of glutathione (GSH) and the expression of Nrf2-dependent antioxidant genes (70).

##### 2.2.1.2 Lipid metabolism

The activation of macrophages is closely related to changes in lipid metabolism. Lipidomic studies have elucidated the importance of lipid metabolism in the polarization of macrophages to an inflammatory phenotype. The synthesis of fatty acids in M1 macrophages is positively regulated to promote proinflammatory functions (71, 72). In LPS-activated BMDMs, the expression of sterol regulatory element-binding protein 1α (SREBP-1α) increases, activating not only fatty acid synthesis but also the Nlrp1α gene, a core component of the inflammasome, which promotes the secretion of proinflammatory cytokines (73). In M1 macrophages, fatty acid synthetase (FAS) is a key factor in regulating fatty acid synthesis, and its absence can prevent chronic inflammation in mice (74). Researchers believe that FAS is necessary for macrophage membrane remodeling; a deficiency in FAS leads to changes in cell membrane components and alterations in the transport of Rho GTPases, thereby reducing inflammatory signaling in macrophages (75). The preference of M1 macrophages for fatty acid synthesis may be related to their pro-inflammatory functions and the regulation of signaling pathways. However, M2 macrophages primarily utilize OXPHOS and fatty acid oxidation (FAO) as their main functional pathways, engaging in activities related to tissue damage repair (76). Adenosine monophosphate-activated protein kinase (AMPK) is a key energy-sensing enzyme, which can be regulated by multiple factors, including the ATP/AMP ratio, phosphorylation by upstream kinases (such as LKB1 and CaMKKβ), and metabolic stress signals (77). The AMPK signaling pathway is activated in the M2 subtype and is involved in increasing OXPHOS and FAO (78). AMPK activation is important for macrophage polarization. For instance, IL-25 induces mitophagy by activating AMPK and promoting the polarization of macrophages toward the M2 phenotype (79). Meanwhile, quercetin inhibits inflammation associated with M1 macrophage polarization and promotes M2 macrophage polarization by activating the AMPK and Akt signaling pathways (80). In hyperhomocysteinemia, intermedin can rescue the imbalance of the M1/M2 macrophage ratio by activating the AMPK pathway (81). Wang et al. (82) discovered that endosome-associated trafficking regulator 1 (ENTR1) can inhibit M1 macrophage polarization by enhancing AMPK phosphorylation. Interestingly, recent studies found that FAO may also contribute to inflammasome activation. Previous research revealed that NADPH oxidase 4 (NOX4), a source of cellular superoxide anions, induces FAO through carnitine palmitoyltransferase 1A (CPT1A) during NLR family pyrin domain-containing 3 (NLRP3) inflammasome activation and helps in the development of the inflammatory macrophage phenotype (76). Through transcriptomics and extracellular flux analysis, it has been found that in an obese environment, adipose tissue macrophages (ATMs) show an increase in both glycolysis and OXPHOS (83). This metabolic feature is dependent on factors originating from adipose tissue. Meanwhile, human macrophages isolated from the adipose tissue of obese individuals also exhibit a similar metabolic profile, which is associated with a pro-inflammatory phenotype (83).

The specific mechanisms by which differences in fatty acid metabolism lead to opposing activation patterns and distinct functions in macrophages need to be investigated further. The proinflammatory and anti-inflammatory phenotypes may influence different metabolic patterns because of their differing dependencies on energy demands and conversion efficiency (66).

##### 2.2.1.3 Amino acid metabolism

Amino acid synthesis, degradation, and interconversion play significant roles in the polarization and function of macrophages. They can be transformed into various physiologically active substances or generate specific chemical groups through appropriate degradation. Arginine metabolism is a key metabolic pathway that regulates macrophage polarization and inflammatory responses (84). In macrophages, the arginine metabolic pathways mediated by iNOS and arginase play important roles in the M1 and M2 processes. iNOS catalyzes the conversion of arginine to NO. M1 macrophages highly express iNOS, which is essential for their inflammatory functions. In contrast, the arginase pathway, which breaks down arginine and thus reduces NO production, is highly expressed in M2 macrophages. This pathway regulates macrophage proliferation and collagen synthesis (66). Glutamine metabolism also plays a crucial role in regulating immune cell functions. Glutamine metabolism is also a key input for the synthesis of NO through arginine, demonstrating the role of glutamine in macrophage cytotoxicity and antibacterial functions (66). Another characteristic of M2 macrophages compared with M1 macrophages is increased glutamine metabolism. Alpha-ketoglutarate produced by glutamine catabolism promotes the epigenetic activation of M2-type genes in macrophages, whereas glutamine is not required for the development of LPS-stimulated M1-type macrophages (69). Recent studies have shown that the inhibition of serine metabolism can enhance the polarization of M1 while inhibiting the polarization of M2 (85). Additionally, the depletion of intracellular citrulline is crucial for the polarization of proinflammatory macrophages and immune responses (86). Researchers have revealed that impaired glutamine catabolism in macrophages exacerbates atherosclerosis. High-throughput transcriptional and metabolic analyses have shown that the phagocytic ability of macrophages depends on the atypical transaminase pathway rather than traditional glutamate dehydrogenase 1 (GLUD1) to support alpha-ketoglutarate-dependent immune metabolism (87).

##### 2.2.1.4 Trace element metabolism

In addition to the aforementioned metabolic processes, the metabolism of certain trace elements also plays a crucial role in the function of macrophages. Macrophages handle most of the iron released from aging and damaged red blood cells, returning it to the circulation or storing it in ferritin within the cytoplasm to maintain iron homeostasis (88). Iron significantly affects macrophage polarization. Generally, with high levels of intracellular iron activating proinflammatory M1 macrophages while reducing the numbers of anti-inflammatory M2 macrophages (89). Over 60 % of iron-related genes are differentially expressed between M1 and M2 macrophages, which may explain why iron levels in M2 macrophages are typically lower than those in M1 macrophages (90). In the non-alcoholic steatohepatitis (NASH) model, Kupffer cells with iron accumulation exhibit a pro-inflammatory and profibrotic phenotype. Iron accumulation activates the MiT/TFE transcription factors, which in turn upregulate the expression of pro-inflammatory and profibrotic genes (91). However, recent studies found that in some cases, iron-rich macrophages also have anti-inflammatory effects. In macrophages infected with Salmonella, restricting intracellular iron appears to change immune function; for example, IFN-γ can increase the expression of the iron exporter ferroportin (Fpn), increasing iron efflux while stimulating the production of TNF-α (88). Conversely, the absence of nitric oxide synthase 2 (Nos2) reduces Fpn, leading to increased iron accumulation in macrophages and the reduced production of cytokines such as TNF-α, IL-12, and IFN-γ (92). The reduction in iron in macrophages diminishes the signaling of TLR4/TRIF-associated adaptor molecules, leading to decreased production of TNF-α, IL-6, and IFN-β in response to LPS stimulation or Salmonella infection (93). MFe^hi^ macrophages are a subset of adipose tissue-resident macrophages characterized by high intracellular iron stores (94). MFe^hi^ macrophages exhibit gene expression profiles associated with anti-inflammatory functions and iron recycling (95). In the obese state, the iron-handling capacity of MFe^hi^ macrophages is impaired, they become more pro-inflammatory. These changes occur concurrently with adipocyte iron overload and reduced adipocyte adiponectin expression (95). The impact of iron metabolism and other trace element metabolisms on macrophage function still requires further investigation.

#### 2.2.2 Mitochondrial function and biogenesis on macrophages

Mitochondria are crucial for the viability of eukaryotic cells, as they play important roles in bioenergetics, metabolism, and signaling (96). Many studies have focused on the impact of mitochondrial function and biogenesis on macrophages.

Mitochondrial fusion and fission can regulate macrophage polarization and function. Ganglioside-induced differentiation-associated protein 1 (GDAP1L1) phosphorylates dynamin-related protein 1 (Drp1), promoting its translocation from the cytosol to mitochondria, leading to mitochondrial fission. This will promote the M1 macrophage polarization. Consequently, the production of cytokines and chemokines is augmented via the MAPK and NF-κB pathways, leading to psoriasis inflammation (97). In contrast, MFN-mediated normal mitochondrial fusion could promote the M2 macrophage phenotype and inhibit the M1 phenotype. According to the study by Li et al. (98), Xuanfei Baidu Formula (XBF) alleviates casp11-dependent NLRP3 inflammasome activation through promoting MFN1-induced mitochondrial fusion, which prevent macrophage polarization to the M1 pro-inflammatory phenotype.

Mitochondrial biogenesis can also regulate macrophage polarization and function. Research indicates that γ-amino butyric acid (GABA) suppresses α-ketoglutarate dehydrogenase (OGDH) activity, lowering succinyl-CoA (SCOA) levels. This reduction diminishes mitochondrial protein succinylation, thereby boosting oxidative phosphorylation efficiency and promoting M2 macrophage polarization (99). Olfactomedin-like protein 3 (OLFML3) protein enhances M2 polarization by promoting immune-responsive gene 1 (IRG1) mitochondrial localization, catalyzing itaconate production, which maintaining mitochondrial membrane potential and mitochondrial reactive oxygen species (mtROS) homeostasis (100). During chronic kidney disease (CKD), the AMPK signaling pathway is inhibited, which result in mitochondrial biogenesis (nuclear respiratory factor 1 and mitochondrial transcription factor A) as well as mitochondrial-specific proteins (cytochrome C and mitochondrial complex IV) downregulated, leading to M1 polarization of macrophages (101).

### 2.3 Effects of metabolism on macrophages

Some cytokines, metabolic intermediates, and metabolites also have interactive effects on the metabolism of macrophages. They may originate from the macrophages themselves, whereas others may originate from the circulation or other cells (Table 2).

**Table 2:** Metabolism-related molecules and their functions.

**Molecules**		**Functions in macrophage**	**References**
Cytokines	IFN-γ	Promote M1 macrophage polarization Enhance glycolysis by promoting the conversion of PFK to uPFK2	(66, 69, 102)
	IL-4	Promote OXPHOS and FAO in M2 macrophages	(66)
	IL-10	Inhibit glycolysis and promote OXPHOS Promote mitophagy, the lack of which can lead to inflammasome activation and IL-1β production	(103)
	TNF-α	Affect the synthesis of NAD^+^	(104)
Metabolic intermediates	Lactate	Inhibit the polarization of M1 macrophages Promote the polarization of M2 macrophages toward an anti-inflammatory and pro-angiogenic phenotype	(105–110)
	Citrate	Inhibit glycolysis, promote fatty acid synthesis and gluconeogenesis	(69, 111–113)
	Itaconate	Affect immune modulation, suppression of inflammation, and promotion of tolerance Related to macrophage polarization	(111, 114–116)
	Succinate	Affects the stability of HIF-1α	(63, 117, 118)
	ROS	Promote metabolic reprogramming of macrophages Affect inflammation	(66, 122, 123)
Metabolites	blood glucose	Promote the polarization of macrophages toward the M1 phenotype Promote inflammation	(160, 161)
	Insulin	Exacerbate macrophage infiltration in adipose tissue; promote glucose metabolism and inhibits glutamine metabolism	(162–164)
	Ketone bodies	β-HB: Promote the polarization of macrophages toward the M2 phenotype AcAc: Aggravate lipid accumulation in macrophages	(166–169)
	Heme	Induce macrophages to release inflammatory factors, promote inflammation	(170–172)
	Blood lipid	Promote the transformation of foam cells and the progression of atherosclerosis	(173–175)
	Bile acids	Promote the polarization of macrophages toward the M2 phenotype	(176)
	Creatinine	Promote the polarization of macrophages toward the M2 phenotype	(177)
	Uric acid	Promote the polarization of macrophages toward the M1 phenotype	(178)

#### 2.3.1 Cytokines

##### 2.3.1.1 IFN-γ

The activation of macrophages to the M1 state through inflammatory stimuli such as IFN-γ is associated with increased glycolytic metabolism (102). The enhancement of glycolytic metabolism is associated with the activation of 6-phosphofructo-2-kinase (PFK 2). Phosphofructokinase-1 (PFK1) is a key enzyme that regulates the flux of glycolysis, and PFK2, the most effective allosteric activator of PFK1, catalyzes the formation of fructose 2,6-bisphosphate. Under stimulation by IFN-γ, the expression of PFK 2 in the liver shifts to a more active, ubiquitously expressed isoform (uPFK2), which helps maintain higher concentrations of fructose 2,6-bisphosphate, thereby enhancing the glycolytic process (66). The increased glycolytic rate allows M1 macrophages to rapidly produce enough energy and biosynthetic intermediates to respond quickly to hypoxic microenvironments (69).

##### 2.3.1.2 IL-4

Unlike those stimulated with IFN-γ, macrophages stimulated with IL-4 primarily undergo glucose metabolism and an energy supply through the TCA cycle and OXPHOS. The TCA cycle in M2 macrophages is involved in the production of uridine diphospho-N-acetylglucosamine (UDP-GlcNAc) intermediates, which are associated with the clearance function of M2 macrophages (66). IL-4 promotes mitochondrial respiration and FAO in M2 macrophages by activating PPARγ coactivator 1β (PGC-1β) (66).

##### 2.3.1.3 IL-10

IL-10 can inhibit LPS-induced glucose uptake and glycolysis in macrophages and promote OXPHOS (103). Furthermore, IL-10 suppresses the activity of the mammalian target of rapamycin (mTOR) by inducing the mTOR inhibitor DDIT4. Consequently, IL-10 promotes mitophagy, which eliminates dysfunctional mitochondria characterized by low membrane potential and high levels of ROS (103). In the absence of IL-10 signaling, macrophages in mouse models of colitis and inflammatory bowel disease accumulate damaged mitochondria, leading to the dysregulation of NLRP3 inflammasome activation and IL-1β production (103).

##### 2.3.1.4 TNF-α

NAD^+^ is an important intracellular redox coenzyme. Studies have shown that TNF-α is related to NAD^+^ levels in macrophages. M1 macrophages stimulated with LPS exhibit biphasic and transient increases in NAD^+^ levels associated with the release of TNF-α (104). TNF-α can regulate the expression of several enzymes involved in NAD^+^ homeostasis (104). TNF-α can regulate the expression of nicotinamide phosphoribosyl transferase (NAMPT), which is a key enzyme in the NAD^+^ salvage pathway (104). By affecting the expression of NAMPT, TNF-α indirectly influences the synthesis of NAD^+^.

#### 2.3.2 Metabolic intermediates

##### 2.3.2.1 Lactate

Lactate, the end product of glycolysis, has long been considered a metabolic waste product (105). However, an increasing body of evidence suggests that lactate has multiple biological functions; it acts as a signaling molecule, regulating various processes such as metabolism, immune responses, and cell–cell communication (106). As a central molecule, lactate plays a crucial role in modulating macrophage metabolism. Studies have shown that lactate can inhibit the polarization of M1 proinflammatory macrophages while promoting the polarization of M2 macrophages toward an anti-inflammatory and proangiogenic phenotype, with these effects being mediated through various mechanisms, including histone posttranslational modifications and signaling pathway regulation (107, 108). Lactate increases phosphorylation activation of the signal-regulated kinase (ERK)/ signal transducer and activator of transcription 3 (STAT3) pathway in macrophages and promotes M2 polarization (109). In 2019, Zhang et al. (110) reported that M1 macrophages possess an endogenous “lactate clock,” which regulates the expression of M2-type characteristics in M1 macrophages during the later stages of polarization through histone lactylation. Some studies have pointed out that, under specific circumstances, lactylation can alter the activity of glycolytic enzymes, thereby inhibiting glycolysis, reducing lactate production, and promoting the shift from M1 phenotype to M2 phenotype (109). The impact of lactate on macrophage polarization remains somewhat controversial and requires further investigation.

##### 2.3.2.2 Citrate

The production and conversion of citrate link mitochondrial and cytoplasmic metabolism. Citrate is generated in the TCA cycle through the condensation of oxaloacetate and acetyl-CoA, with the latter derived from pyruvate produced by glycolysis or the breakdown of fatty acids. Citrate is converted to isocitrate and then transformed into α-ketoglutarate (αKG) by IDH (111). Citrate entering the cytoplasm can indirectly inhibit glycolysis by acting on pyruvate kinase (PK), stimulate fatty acid synthesis by activating acetyl-CoA carboxylase (ACC), and promote gluconeogenesis by activating fructose-1,6-bisphosphatase (111). As previously introduced, the disruption of the TCA cycle in M1 macrophages leads to the accumulation of citrate. Owing to the downregulation of IDH expression and the upregulation of the expression of the mitochondrial citrate carrier (CIC), M1 macrophages characteristically accumulate citrate, which is then transported out of the mitochondria (112). This accumulation and transfer of citrate are crucial for the functions of M1 macrophages, affecting the production of NO, ROS, and prostaglandin E2 (PGE2) in M1 macrophages (112, 113).

##### 2.3.2.3 Itaconate

Itaconate is produced from cis-aconitate in the TCA cycle by the enzyme itaconate decarboxylase 1 (ACOD1). Itaconate has antibacterial properties and may also play a role in immune modulation, the suppression of inflammation, and the promotion of tolerance (114, 115). ACOD1 expression is upregulated in M1 macrophages (111). Reduced levels of itaconate favor M2-like polarization in macrophages, possibly due to the inhibition of SDH, which increases OXPHOS flux (116).

##### 2.3.2.4 Succinate

Succinate is an intermediate product of the TCA cycle, is produced from succinyl-CoA and is a substrate for succinate dehydrogenase (SDH). As previously noted, the disruption of the TCA cycle in M1 macrophages also results in succinate accumulation. Succinate affects the stability of HIF-1α by inhibiting prolyl hydroxylase domain enzymes (PHDs), which are a class of αKG-dependent dioxygenases that can regulate HIF-1α stability in an oxygen-dependent manner, preventing its degradation (63). In the absence of ATP production, the substantial oxidation of succinate can lead to reverse electron transport (RET) to complex I, which is associated with a significant release of ROS and can activate HIF1-α in M1 macrophages (117, 118). HIF-1α can then interact with coactivators to induce the glycolytic metabolic program, and drive inflammation by increasing transcription of IL-1β (119). Although succinate is primarily associated with the pro-inflammatory responses of M1 macrophages, studies have also shown that it can promote the polarization of M2 macrophages through the succinate receptor-1(SUCNR-1) mediated signaling pathway (120). The impact of succinate on macrophage polarization and function is worth further investigation.

##### 2.3.2.5 ROS

Elevated levels of ROS and hypoxia promote metabolic reprogramming in macrophages, making the cells more reliant on glycolytic processes (66). M1 macrophages generate ROS through the NADPH oxidase complex (such as NOX2) by catalyzing the one-electron reduction of oxygen, which is crucial for macrophage phagocytosis and antimicrobial defense (121). The formation of a high-concentration ROS microenvironment is accompanied by the upregulation of the expression of inflammatory markers such as iNOS, leading to increased glucose uptake and enhanced glycolytic metabolism (122). NO is produced by iNOS, which catalyzes the conversion of arginine and is involved in the proinflammatory response of macrophages (123).

#### 2.3.3 Sirtuins

Sirtuins (SIRT), initially identified in yeast as the first nicotinamide adenine dinucleotide (NAD)^+^-dependent epigenetic and metabolic regulators, belong to the class III histone deacetylases. Their activity relies on the presence of NAD^+^ (124). The sirtuins protein family primarily comprises seven members. SIRT1, SIRT6, and SIRT7 are typically localized in the nucleus, where they are involved in chromosomal stability and transcriptional regulation; SIRT2 is mainly found in the cytoplasm but can also be present in the nucleus; SIRT3, SIRT4, and SIRT5 are usually located in the mitochondria, where they regulate metabolic enzymes and stress response mechanisms (125). The substrates of nuclear sirtuins include histones and non-histone proteins, such as nuclear transcription factors and cofactors. In contrast, cytoplasmic and mitochondrial sirtuins play crucial roles in key enzymes involved in oxidative and metabolic pathways, including glycolysis, fatty acid oxidation, and the TCA cycle (126).

Sirtuins influence macrophage polarization through multiple pathways. SIRT1/2 can directly inhibit the NF-κB signaling pathway by deacetylating the p65 subunit of NF-κB, reducing the expression of pro-inflammatory cytokines (such as IL-6, IL-1β, and TNF-α) and suppressing M1 polarization (127–129). SIRT1 can also upregulate the expression of STAT6 to promote M2 polarization (130). SIRT3 promotes M2 polarization by activating mitochondrial autophagy, which reduces the release of inflammatory cytokines like IL-1β and TNF-α (131, 132). SIRT4 decreases M1 polarization by inhibiting STAT3 and suppressing inflammatory factors such as ROS, MMP-13, IL-6, and TNF-α. Meanwhile, SIRT4 promotes M2 polarization, increases the mRNA levels of Arg-1 and CD206 and enhances the expression of the IL-10 (133, 134). SIRT5 promotes the acetylation of p65 and activates the NF-κB signaling pathway to augment IL-1β production during inflammation (135, 136). SIRT7 activates the NF-κB signaling pathway, increases the expression of IL-1β, and promotes inflammatory responses (137). In contrast, SIRT6 upregulates the expression of anti-inflammatory cytokines IL-4 and IL-10, increases M2 polarization, and attenuates inflammatory responses (138).

The pre-activation state of macrophages also affects the function and expression of Sirtuins. NAD^+^ is an important cofactor regulating metabolic homeostasis and a rate-limiting substrate for sirtuin deacylases (139). Studies have shown that supplementation with NMN (a precursor of NAD^+^) increases NAD^+^ levels and activate SIRT3 (140). Under pro-inflammatory conditions, M1 macrophages produce large amounts of ROS through glycolysis, which may lead to the depletion of NAD^+^ and indirectly inhibit the enzymatic activity of SIRT1 and SIRT3 (59, 141, 142). In contrast, M2 macrophages rely on oxidative phosphorylation and may maintain higher NAD^+^ levels through fatty acid oxidation and OXPHOS, thereby activating SIRT3 and promoting anti-inflammatory and tissue repair functions (59, 141, 143, 144).

#### 2.3.4 NLRP3 inflammasome

The NLRP3 inflammasome is a high-molecular-weight multiprotein complex composed of NLR family members that assembles in response to various stimuli in the cytoplasm (145); it plays a key role in host defense, inflammation, autoimmunity, and the development of metabolic diseases (146). Macrophages undergo profound metabolic reprogramming when they are sensing infections and sterile stimuli, and this metabolic shift supports and regulates the activation of the NLRP3 inflammasome (147).

The role of glycolysis in inflammasome activation is complex and involves enzymes involved in the glycolytic process and changes in glycolytic flux. The blockade of pyruvate kinase M2 (PKM2) limits the classical activation of NLRP3 in mouse BMDMs (148). In contrast, the inhibition of hexokinase 2 (HK2) by N-acetylglucosamine (NAG) leads to the detachment of HK2 from the mitochondrial outer membrane, thereby increasing inflammasome activation in mouse BMDMs (149). Effective glycolysis results in the conversion of NAD^+^ to NADH, the secretion of lactate, and the production of ATP. The inhibition of GAPDH and α-enolase, leading to the interruption of glycolytic flux, triggers the NLRP3 inflammasome in mouse BMDMs in a K^+^-independent manner (150).

Intermediates in the TCA cycle play a significant role in cellular signaling, including the regulation of NLRP3 inflammasome activation. The accumulation of itaconate is associated with the inhibition of the NLRP3 inflammasome. *Irg1*^−/−^ mouse BMDMs, which cannot synthesize itaconate, exhibit increased NLRP3 activation (114). Itaconate inhibits the activation of the NLRP3 inflammasome through various mechanisms, including posttranslational modifications of kelch-like ECH-associated protein 1 (KEAP1), NIMA-related kinase 7 (NEK7), and gasdermin-D (GSDMD) (151–155). Succinate plays a significant role in regulating NLRP3 inflammasome activation by succinylating the Cys192 site of GSDMD, inhibiting GSDMD oligomerization and pyroptosis (156).

Lipid metabolism is also crucial for NLRP3 inflammasome production. Saturated fatty acids (SFAs) can activate the NLRP3 inflammasome, whereas unsaturated fatty acids, such as oleic acid (OA), can counteract SFA-mediated NLRP3 activation or directly suppress NLRP3 activation (155, 157–159). Polyunsaturated fatty acids (PUFAs), such as docosahexaenoic acid (DHA), can sense and inhibit LPS-preactivated human macrophage NLRP3 activation (151, 152).

#### 2.3.5 Metabolites

##### 2.3.5.1 Blood glucose

Chronic hyperglycemia enhances the proinflammatory response in lipopolysaccharide-stimulated macrophages, including the production of TNF-α, IL-1β, and IL-6 (160). Studies have also shown that under high-glucose culture conditions, BMDMs and monocytes exhibit increased glycolysis, altered metabolic profiles, and a shift toward proinflammatory subtype (M1 type) polarization, promoting the expression of proinflammatory genes in macrophages. This may explain how hyperglycemia induces trained immunity in macrophages and their precursor cells (161).

##### 2.3.5.2 Insulin

Insulin also exerts an impact on the metabolism and function of macrophages. Studies have shown that in patients with type 2 diabetes, the number of macrophages in adipose tissue and the circulating levels of TNF-α and IL-1β increase following the initiation of insulin therapy (162). Hyperinsulinemia can exacerbate the infiltration of macrophages into adipose tissue and promote the development of insulin resistance (163). A previous study revealed that insulin enhances the phagocytic capacity of macrophages and the production of hydrogen peroxide (H_2_O_2_) by promoting glucose metabolism and inhibiting glutamine metabolism, thereby strengthening their immune function (164).

##### 2.3.5.3 Ketone bodies

Ketone bodies (KBs), which include β-hydroxybutyric acid (β-HB) and acetoacetic acid (AcAc), play critical roles in organismal energy homeostasis (165). Studies have shown that in atherosclerosis, β-HB inhibits Ox-LDL-induced macrophage proliferation (166). AcAc inhibits proliferation but exacerbates lipid accumulation (166). These two may exert opposite effects on atherosclerotic progression. β-HB could promote M2 macrophage polarization through the STAT6-dependent signaling pathway, and suppress M1 macrophage polarization through β-hydroxybutyrylation of the STAT1 protein (167, 168). When rats were treated with a ketone diet, it was found that ketogenic metabolism could promote the polarization of macrophages toward the M2 phenotype and inhibit inflammatory responses (169).

##### 2.3.5.4 Heme

Heme is an early important substance in the activation of innate immunity and has been shown to directly act on macrophages, neutrophils, epithelial cells, and endothelial cells (170). As a typical proinflammatory response molecule, heme can directly induce macrophages to produce proinflammatory lipid mediators such as leukotriene B4 (LTB4) and release proinflammatory cytokines, leading to local to systemic inflammatory responses (171). Studies have also indicated that heme can stimulate NLRP3 inflammasome activation in endothelial cells, inducing macrophages to produce large amounts of IL-1β, which often leads to inflammatory responses in hemolytic diseases (172).

##### 2.3.5.5 Blood lipids

The impact of blood lipids on macrophages is crucial in various types of inflammation. Mice fed a high-fat diet (Western diet) present increased levels of cholesterol and triglycerides, with the activation of endoplasmic reticulum stress pathways in cardiac macrophages leading to the activation of inflammatory genes and affecting myocardial inflammation in heart failure with preserved ejection fraction (173). Monocyte-derived macrophages can take up oxidized low-density lipoprotein in the subendothelial space of blood vessels, leading to the accumulation of cholesterol and the formation of foam cells, which is a hallmark of atherosclerotic disease progression (174). In contrast to LDL, high-density lipoprotein (HDL) inhibits the expression of surface markers and inflammation-related genes such as TNF-α, IL-6, and monocyte chemotactic protein-1 (MCP-1) in macrophages, reducing the production of ROS and RNS and blocking the polarization of macrophages toward the M1 type (175).

##### 2.3.5.6 Bile acids

Bile acids (BAs) are steroid substances synthesized from cholesterol in the liver that are capable of binding to plasma proteins and entering the systemic circulation, where they are distributed to various organs throughout the body (such as the intestines, brain, and heart). BAs affect the polarization state of macrophages by activating different receptors, such as the farnesoid X receptor (FXR) and the G protein-coupled bile acid receptor 1 (TGR5) (176). The activation of TGR5 can reduce the secretion of TNF, IFN-γ, IL-6, and IL-1β in proinflammatory M1 macrophages and promote the polarization of anti-inflammatory M2 macrophages (176).

##### 2.3.5.7 Creatinine

Creatinine is a slightly toxic metabolic product produced by muscle metabolism and is primarily excreted by the glomerular filtration of the kidneys, and its levels significantly increase after extensive exercise or the excessive consumption of meat. Macrophages mainly take up creatinine through solute carrier family 6 member 8 (SLC6A8), and subsequently, creatinine inhibits the IFN-γ/JAK/STAT1 signaling pathway, reducing the expression of iNOS, IL-12, and TNF-α and blocking the polarization of macrophages toward the M1 type. On the other hand, creatinine can promote chromatin remodeling and activate IL-4/STAT6 signaling, promoting the expression of M2 markers such as Arg-1 (177).

##### 2.3.5.8 Uric acid

Uric acid is the end product of purine metabolism, and disturbances in purine metabolism often lead to abnormally high levels of blood uric acid, causing hyperuricemia, which is closely related to the occurrence of gouty arthritis, joint deformity, and gouty nephropathy. Studies have shown that uric acid significantly increases the expression of TNF-α and TLR4 in macrophages and enhances their phagocytic activity, while the expression levels of CD206, C-X3-C motif chemokine receptor 1 (CX3CR1), and C-C chemokine receptor 2 (CCR2) are significantly reduced, leading to M1-type polarization and the downregulation of urate transporter 1 (URAT1) expression (178). Probenecid treatment significantly inhibits UA-induced M1 macrophage polarization, reducing the production of proinflammatory cytokines and weakening their phagocytic activity (178).

## 3 The role of macrophages in inflammatory heart disease

### 3.1 Macrophages in the heart

#### 3.1.1 Classification and function of macrophages in the heart

In the cardiac tissue microenvironment, macrophages constitute the largest group of immune cells. Cardiac macrophages can be divided into two major categories: infiltrating macrophages and resident macrophages.

Infiltrating cardiac macrophages are mainly CCR2^+^ macrophages derived from recruited monocytes, which play a major role in the inflammatory response (179). After myocardial infarction, macrophages and other inflammatory cells infiltrate the infarct area, leading to the production of pro-inflammatory cytokines, exacerbating inflammation, and clearing necrotic tissue (180). In ischemic myocardial injury, CCR2^+^ macrophages infiltrated via MCP-1 dominate the inflammatory and fibrotic responses in the early stage of injury (181). Additionally, in dilated cardiomyopathy, NLRP3 inflammasomes synthesized by infiltrating macrophages can promote the cleavage of apoptosis-associated speck-like protein (ASC), caspase-1, IL-1β, IL-18, and GSDMD into their active forms, which will exacerbate inflammation, induce cardiomyocyte pyroptosis and promote myocardial fibrosis (182, 183).

Resident cardiac macrophages originate from different macrophage precursor cells and possess distinct functions and behaviors (184). Resident cardiac macrophages in the heart can be distinguished on the basis of their expression of CCR2; CCR2^−^ macrophages are derived from the primitive yolk sac and fetal monocyte precursors, whereas CCR2^+^ macrophages originate from hematopoietic progenitors in the blood (185, 186).

Under homeostatic conditions, resident cardiac macrophages are involved in a range of functions that are crucial for maintaining cardiac homeostasis. Studies have shown that CCR2^−^ macrophages, which originate from the yolk sac, are necessary for the proper remodeling of the coronary arterial plexus and the functional maturation of coronary vessels (187). These findings also suggest that macrophage-derived insulin-like growth factor 1 (IGF-1) is a potential mediator of coronary arteriogenesis (187). CCR2^−^ macrophage depletion in embryos leads to abnormalities in developmental processes and vascular maturation (187).

Resident cardiac macrophages mediate immune surveillance, which maintains the homeostasis of CCR2^−^ and CCR2^+^ macrophages that express MHC II and act as antigen-presenting cells; they function by processing and presenting antigens to circulating T cells (184). Moreover, CCR2^−^ resident cardiac macrophages expressing MERTK have a greater capacity for phagocytosing cardiac myocyte debris and contents than CCR2^+^ macrophages do. Several studies have described the efferocytosis of apoptotic cells by MERTK-expressing cardiac macrophages as an important contributing factor to cardiac homeostasis (188, 189). Additionally, MERTK-expressing resident cardiac macrophages in the hearts of young mice can protect the heart from infection by *Mycobacterium avium*, which has been shown to cause cardiac arrhythmias and promote bacterial dissemination to the cardiac tissues of older mice (190).

Importantly, studies have shown that resident cardiac macrophages promote mitochondrial homeostasis in cardiomyocytes (191). Mertk deletion in resident cardiac macrophages limits their phagocytic capacity and prevents them from taking up dysfunctional mitochondria (191). The disruption of mitochondrial clearance in macrophages impairs autophagy, leading to the accumulation of cellular debris and metabolic dysfunction in cardiomyocytes, which may result in impaired ventricular function (191).

With the development of technologies such as single-cell RNA sequencing (scRNA-seq) and spatial transcriptomics, further classification of cardiac macrophage subsets has been achieved. Using scRNA-seq, trypanosome lytic factor (TLF^+^) macrophages have been identified (192–194). The replenishment of TLF^+^ macrophages is independent of circulating monocytes (192), and their transcriptomic features are mainly enriched in homeostatic functions, such as endocytosis, cellular transport, and angiogenesis (192, 193). The application of scRNA-seq in acute myocardial infarction research has further subdivided distinct subsets of cardiac macrophages. It was found that macrophages with high expression of interferon-stimulated genes (ISG^+^) and MHC-II^+^ macrophages are key pro-inflammatory subsets during the inflammatory phase, whereas macrophages with high expression of triggering receptor expressed on myeloid cells 2 (Trem2^+^) are the main anti-inflammatory subsets during the repair phase (194–196). A study using spatial transcriptomics analyzed different anatomical regions of the heart and identified a novel population of tissue-resident macrophages (LYVE1^+^ macrophages), which are present in the atrioventricular node and sinoatrial node (197). Another study utilizing spatial transcriptomics discovered a subset of monocyte-derived macrophages expressing basic helix-loop-helix family member e41 (Bhlhe41^+^) in the infarct area during the early stage of myocardial infarction (198). By analyzing spatial ligand-receptor interactions and combining experiments in animal models, the results showed that Bhlhe41^+^ macrophages can upregulate the secretion of progranulin (GRN). GRN antagonizes the effect of TNF-α on TNF receptor 1 (TNFR1), which will inhibit myofibroblast activation and limit the expansion of the infarct area (198). In the future, with the continuous advancement of technologies and in-depth research, the classification of cardiac macrophages will be further refined.

#### 3.1.2 Crosstalk between cardiac macrophages and other cells

Cardiac macrophages participate in cross talk with fibroblasts, lymphocytes, endothelial cells, cardiomyocytes, and other cell types in the heart (199). Following myocardial infarction, pro-inflammatory M1-type macrophages release exosomes rich in miR-155, which suppress the expression of key genes such as Rac family small GTPase 1 (RAC1), p21-activated kinase 2 (PAK2), SIRT1, and AMP-activated protein kinase α2 (AMPKα2) in endothelial cells. This inhibition of gene expression could impair angiogenesis and exacerbates myocardial injury (200). During the healing phase of myocardial infarction, infiltrating cardiac macrophages can secrete Angiotensin II (Ang II), which activates the angiotensin II type 1 (AT1) receptor on fibroblasts, upregulates TGF-β1, induces the transformation of fibroblasts into myofibroblasts and increases collagen deposition (201). Meanwhile, infiltrating cardiac macrophages are capable of secreting exosomes enriched with miR-155; after being internalized by fibroblasts, these exosomes downregulate the expression of son of sevenless homolog 1 (SOS1) and inhibit fibroblast proliferation (202). Interestingly, the expression of angiopoietin 2 (Angpt2) in endothelial cells is increased, and Angpt2 can promote the polarization of macrophages toward a pro-inflammatory phenotype via the α5β1/extracellular ERK pathway (203). Similarly, after myocardial infarction, neutrophil gelatinase-associated lipocalin (NGAL) in the secretome of neutrophils affects the proliferation of cardiac macrophages by modulating the expression of the macrophage receptor MertK on the cardiac macrophage membrane (204). In the absence of NGAL, the expression of MertK on the cardiac macrophage membrane is reduced, which impairs the phagocytic capacity of macrophages (204). Th1 cells promote the polarization of pro-inflammatory macrophages, whereas Th2 cells and Treg cells (which secrete IL-10 and TGF-β) facilitate the acquisition of an anti-inflammatory macrophage phenotype. These regulatory effects ultimately influence myocardial healing and scar formation (205). A pioneering study reported that resident cardiac macrophages promote electrical conduction in the heart by intermingling with distal atrioventricular nodal conduction cells through macrophages expressing connexin 43 (206). Both mouse and human hearts are rich in CCR2^−^ and CCR2^+^ macrophages in the atrioventricular node (206). Resident cardiac macrophages in the atrioventricular node maintain direct contact with cardiomyocytes through gap junctions. The removal of resident cardiac macrophages or conditional knockout of the Gja1 gene, which encodes connexin 43, leads to impaired atrioventricular conduction, ultimately resulting in atrioventricular block, thereby confirming that resident cardiac macrophages are necessary for cardiac electrical conduction. Furthermore, compelling studies have indicated that sudden cardiac death due to arrhythmias caused by myocardial infarction is a result of an imbalance in the cardiac leukocyte population (207, 208). More specifically, researchers have described an “electrical storm,” during which rapidly infiltrating neutrophils contribute to ventricular tachycardia (207, 208). The depletion of CCR2^−^ resident cardiac macrophages or complete depletion of macrophages leads to an increased incidence of arrhythmias, demonstrating that macrophages have a protective effect on electrical conduction after myocardial infarction.

### 3.2 Macrophages function in inflammatory heart disease

**Figure 3:**
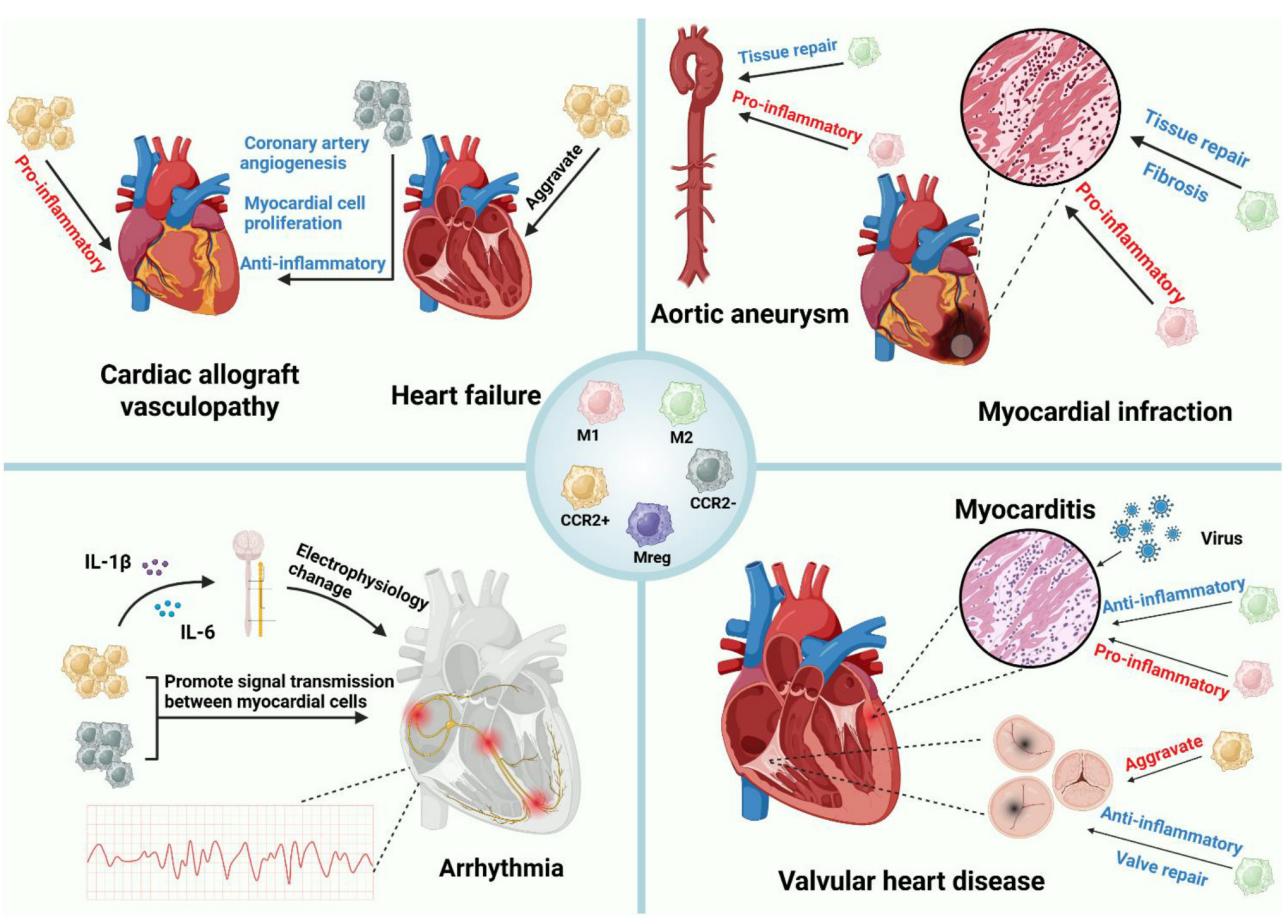
Macrophages in inflammatory heart disease. Macrophages play different roles in various heart diseases. In atherosclerosis, the recruitment, polarization, and foam cell formation of macrophages affect plaque formation. After MI, cardiac macrophages play different roles at various stages, including pro-inflammatory functions, clearance of necrotic cells, and tissue repair. In aortic aneurysms, M1 macrophages drive the inflammatory process, while M2 macrophages promote vascular remodeling and repair. In myocarditis, M1 macrophages exacerbate the condition, while M2 macrophages alleviate inflammation. CCR2^+^ macrophages promote inflammatory responses in transplant rejection, whereas CCR2^−^ macrophages have anti-inflammatory effects and promote coronary artery formation and cardiomyocyte proliferation. In valvular heart disease, CCR2^+^ macrophages are associated with disease progression, while M2 macrophages can reduce inflammation and promote valve repair. The proliferation of CCR2^+^ macrophages is associated with the deterioration of cardiac function in heart failure. Myocardial macrophages are crucial for maintaining normal cardiac electrical conduction, and the inflammatory factors they secrete are closely related to arrhythmias (figure was created with Biorender.com).

Macrophages not only are crucial for maintaining cardiac homeostasis but also play a significant role in heart diseases (Table 3). Research has shown that, in heart-related diseases such as myocardial infarction, myocarditis, valvular heart disease, and heart failure, macrophage polarization, metabolic changes and functions are important (Figure 3).

**Table 3:** Key molecules and pathways of macrophage actions in inflammatory heart diseases.

**Disease**	**Key molecules**	**Important functions and molecular pathways**	**References**
Atherosclerosis	IL-1, IL-6, TNF-α, oxLDL, Adhesion molecules, Chemokines	Macrophage polarization (M1/M2), foam cell formation, inflammatory response	(66, 209–221)
Myocardial infarction	DAMPs, TLRs, HIF-1α, HK1, LDH, GLUT1, SIRT1, Pbx1, IL-10, TREM2, SYK, SMAD4, SLC25A53, Itaconate	TLR-mTOR-HIF1α axis, SIRT1-Pbx1-IL-10 pathway, SYK-SMAD4-SLC25A53-itaconate axis	(111, 119, 180, 184, 222–233)
Myocarditis	IL-1β, NLRP3, iNOS, IL-6, IL-13, IL-10, IFN-α, TGF-β, IL-4, GM-CSF, IL-17, Prominin-1/CD133, VEGF, MMPs, TIMPs	M1/M2 polarization, NLRP3 inflammasome activation, GM-CSF/IL-6/Th17 positive feedback loop	(234–249)
Heart allograft rejection	CCR2, IL-10, TGF-β, MEK1/2, PKM2	MEK1/2-PKM2 pathway	(17, 28, 250–258)
Valvular heart disease	TGF-β1, TNF-α, IL-1β, IL-2, CCR2, CD206, TLR7, IL-10, STAT3β	TLR7-IL-10 pathway, STAT3 alternative splicing	(259–265)
Heart failure	TNF-α, IL-6, IL-1β, Ly6C, HIFs, MMPs, Galectin-3, TGF-β	HIF-glycolysis (Slc2a1), NLRP3 inflammasome activation	(266–278)
Arrhythmias	CRP, hs-CRP, IL-6, IL-1β, TNF-α, CCR2, Connexin 43, SPP1	Gap junction disruption, Sympathetic nervous system activation	(279–285)
Aortic aneurysm	CCR2, ROS, TNF-α, IL-1β, IL-10, TGF-β, MMPs, KLF6, PPARδ	M1/M2 polarization, Oxidative stress, ECM degradation	(197, 286–295)

#### 3.2.1 Atherosclerosis

Atherosclerosis (AS) is a process characterized by the formation of fibrofatty lesions in the intima of arteries, a term derived from Greek, meaning “gruel-like,” which vividly describes the lipid core of typical atherosclerotic plaques (209, 210). Over time, atherosclerotic plaques become more fibrotic and accumulate calcium (209). Plaques can trigger thrombosis and cause tissue ischemia by obstructing blood flow through rupture, superficial erosion, or protrusion into the arterial lumen (211). Inflammation plays a crucial role in AS development. Atherosclerotic plaques harbor a variety of inflammatory cells, such as monocytes/macrophages and T cells. These cells are recruited to sites of arterial wall injury, where they release a range of inflammatory factors, including cytokines (IL-1, IL-6, TNF-α, etc.), which contribute to the inflammatory response (212, 213). After engulfing oxidized low-density lipoprotein (oxLDL), monocytes/macrophages transform into foam cells, and the accumulation of foam cells is one of the key features of atherosclerotic plaque formation (214).

Macrophages are involved in the occurrence, development, and regression of AS. The recruitment and polarization of macrophages and the formation of foam cells can affect the fate of plaques (66). When the endothelium is exposed to atherogenic risk factors, endothelial cells are disturbed. These risk factors include oxidized and other modified LDL particles, pro-inflammatory cytokines, local disturbances in the hemodynamic environment, and cardiovascular-related risk factors (215, 216). Activated endothelial cells express adhesion molecules and chemokines, which promote the rolling, adhesion of circulating monocytes to the endothelium, and the migration of bound monocytes into the arterial wall (217).

Histological analyses of human plaques have revealed varying numbers and degrees of macrophages. M1 macrophages are located primarily in lipid-rich areas away from M2 macrophages; these macrophages change their phenotypic expression over time, depending on their location and microenvironment, all of which regulate plaque progression and composition (66). Macrophages take up oxidized lipid particles and transform into cholesterol-filled foam cells, which is an early event in plaque formation. The formation of foam cells is associated with multiple transcription factors that play key roles in macrophage polarization and the development of atherosclerosis (218). The dynamic equilibrium of macrophages within atherosclerotic plaques is a complex process involving multiple aspects, including recruitment, retention, polarization, egression, and death. The interplay of these processes determines the inflammatory status and stability of the plaque (219). In the early stages of atherosclerosis, the dominant M2 macrophages help maintain the stability of the arterial wall (220). However, as the disease progresses, the proportion of M1 macrophages gradually increases, while the proportion of M2 macrophages relatively decreases. This imbalance exacerbates the inflammatory response and promotes the development of atherosclerosis (221).

#### 3.2.2 Myocardial infarction

Myocardial infarction (MI) is a clinical condition characterized by cardiomyocyte death and tissue injury resulting from myocardial oxygen deprivation. The loss of cardiomyocytes is accompanied by alterations in cellular composition, which in turn elicits an inflammatory response and triggers remodeling of the ischemic myocardium (222). Various cells, including cardiomyocytes, endothelial cells, neutrophils, monocytes/macrophages, lymphocytes, and fibroblasts, are involved in the inflammatory response. These cells promote the infiltration of inflammatory cells and the clearance of tissue damage by releasing cytokines, chemokines, and cell adhesion molecules (180). MI is one of the leading cardiovascular diseases worldwide, and its high incidence and mortality rates pose a significant burden on society and healthcare systems (223). Immune inflammation plays a crucial role in MI and subsequent ventricular remodeling, significantly impacting the extent of tissue damage, cardiac function, and disease prognosis (224). As essential innate immune cells, macrophages play a vital role in immune responses, pathogen clearance, and tissue repair (225). After MI, there are significant changes in the subpopulation distribution, metabolic characteristics, and functional phenotypes of cardiac macrophages, which promote inflammation, dead cell debris clearance, inflammation/repair transition, tissue repair promotion, and other functions at different stages (184, 226).

The insufficient cardiac blood perfusion after MI leads to a lack of oxygen and nutrients, cell damage, and the release of DAMPs. These environmental changes trigger adaptive alterations in macrophage metabolic pathways, significantly affecting their movement, phagocytic functions, and cellular phenotypes, thereby influencing the role of macrophages in disease occurrence and development (111, 224). Transcriptome profiling has revealed differential gene enrichment in glycolytic pathways in macrophages on the first day post-MI; metabolic flux analysis has revealed that macrophage glycolysis increases on the Day 1 and Day 3 post-MI. Enhanced glycolysis in macrophages early after MI is conducive to their adaptation to hypoxic environments and activation (227). Mechanistically, macrophages post-MI recognize DAMPs released from necrotic cells through Toll-like receptors (TLRs), activating downstream signaling pathways such as the mTOR pathway and entering an activated state with increased energy demands (224, 226). Hypoxia-induced factor (HIF)-1α, a downstream molecule of the TLR–mTOR axis, increases in expression post-MI and enhances glycolysis by upregulating the expression of key enzymes in the glycolytic pathway, such as hexokinase 1, lactate dehydrogenase, and glucose transporter 1 (228). In a mouse MI model, the double knockout of HIF-1α and HIF-2α reduces macrophage glycolytic levels, leading to increased macrophage necrosis, impaired tissue fibrosis, and increased cardiac rupture in mice, suggesting that increased macrophage glycolysis post-MI is beneficial for their survival in hypoxic environments (229). Additionally, the enhancement of glycolysis in macrophages post-MI provides energy for cell movement, phagocytosis, and the secretion of inflammatory factors while reducing oxygen consumption, meeting their increased energy demands upon activation (119, 184, 226).

To date, there is a lack of research evidence on lipid synthesis metabolism in macrophages post-MI. Some studies suggest that macrophages enhance FAO when phagocytosing necrotic cells post-MI, activating NAD^+^-dependent signal transduction and promoting the SIRT1–Pbx1–IL-10 metabolic signaling pathway, which promotes macrophage polarization toward the M2 type and improves cardiac damage repair post-MI (230). The changes in amino acid metabolism in macrophages post-MI have also been less studied. Glutamine metabolism in macrophages increases post-MI in mice, and exogenous glutamine supplementation can improve left ventricular function on the first, third, and seventh days post-MI (231). Recent studies have found that during the subacute phase of myocardial infarction, TREM2^+^ macrophages are recruited to the infarcted myocardial tissue and show upregulation of anti-inflammatory genes (232). These cells, derived from myeloid cells, play an important role in myocardial infarction repair (233). After efferocytosis, TREM2^+^ macrophages inhibit the mitochondrial NAD^+^ transporter SLC25A53 via the SYK-SMAD4 signaling pathway. This affects mitochondrial NAD^+^ transport, interrupts the TCA cycle, and increases itaconate production. Itaconate, an anti-inflammatory metabolite, helps regulate macrophage function and cardiac repair after myocardial infarction (233).

#### 3.2.3 Myocarditis

Myocarditis is an inflammatory heart disease that may be triggered by infections, exposure to toxic substances, and activation of the immune system. It is classified as a secondary cardiomyopathy in the 1996 World Health Organization classification (234). The most thoroughly studied to date is enterovirus infection, particularly myocarditis caused by Coxsackie virus B3 (CVB3) infection (235). The mechanisms by which this virus causes myocardial injury include both direct viral damage and immune-mediated damage. The innate immune response of the host is excessively activated (236, 237), leading to the release of a large number of inflammatory factors and the formation of a cytokine storm, which is the main mechanism causing severe myocardial injury and pump failure (238).

Research on the role of macrophages in myocarditis has focused primarily on the M1 and M2 polarization phenotypes, with a general consensus that M1 macrophages exacerbate myocarditis, whereas M2 macrophages alleviate myocardial inflammation. In viral myocarditis caused by CVB3, macrophages are enriched in cardiac tissue as early as the third day post infection (239), and they secrete high levels of IL-1β, which triggers the upregulation of NLRP3 in macrophages, potentially polarizing them to the M1 phenotype (240, 241). M1 macrophages are characterized by enhanced glycolysis and disrupted TCA cycle, leading to the accumulation of HIF-1α and the expression of pro-inflammatory factors (iNOS, IL-1β, IL-6) (242). This further exacerbates inflammation, creating a vicious cycle. The adoptive transfer of M1 macrophages significantly promotes myocarditis in mice. Transferring M2 macrophages into susceptible male mice significantly reduces myocardial inflammation by modulating local cytokines (243). Similarly, in myocarditis induced by CVB3, IL-13 deficiency increases the severity of CVB3-induced myocarditis by reducing the number of M2 macrophages but does not affect viral replication in the heart postinfection. The reduction in M2 macrophages leads to a significant increase in the levels of IL-1, IL-18, IFN-α, TGF-β, IL-4, and histamine, as well as an increase in CD4^+^ T cells and autoreactive antibodies (244). In the experimental autoimmune myocarditis (EAM) model, macrophages are one of the major cellular components of cardiac inflammation. Studies have shown that they are not only key players in the inflammatory response but also regulate disease progression and tissue repair through interactions with different immune cells (245). Granulocyte-macrophage colony-stimulating factor (GM-CSF) stimulates macrophages to secrete IL-6, which in turn promotes the development of Th17 cells and enhances autoimmune reactions. The IL-17 secreted by Th17 cells can further activate macrophages, forming a positive feedback loop (246). Prominin-1^+^/CD133^+^ cells (a type of hematopoietic cell with stem cell characteristics) can differentiate into macrophages with immune regulatory functions in EAM. These macrophages can secrete anti-inflammatory cytokines (such as IL-10) to suppress inflammatory responses, alleviating the severity of myocarditis (247). IL-13 alleviates the severity of myocarditis by regulating the polarization and function of monocytes/macrophages. Its absence may lead to enhanced T cell activation and altered macrophage activation states, resulting in exacerbated myocarditis, cardiac fibrosis, and heart failure (244). During the chronic phase of myocarditis, Ly6C^high^ monocytes infiltrated the heart transition into Ly6C^low^ macrophages, while Ly6C^low^ monocytes continue to infiltrate the heart and differentiate into macrophages (248). These macrophages predominantly exhibit an M2 phenotype and secrete a variety of cytokines that mediate tissue repair: arginase 1, TGF-β, and IL-10 induce myofibroblasts to produce collagen, VEGF promotes angiogenesis, and MMPs along with tissue inhibitors of metalloproteinases (TIMPs) regulate the extracellular matrix network. In contrast, TNF-α and IL-1 secreted by M1 macrophages stimulate fibroblasts to secrete excessive extracellular matrix, leading to matrix deposition and maladaptive fibrosis (249).

#### 3.2.4 Heart allograft rejection

Inflammation in heart allograft rejection is one of the important factors leading to transplant failure, including hyperacute rejection, acute rejection, and chronic rejection. Hyperacute rejection is an immune response that occurs rapidly, usually within minutes to hours after transplantation. The inflammatory manifestations include vascular congestion, thrombosis, hyaline microthrombi, and necrotizing vasculitis of small to medium-sized vessels (250). Acute rejection includes acute cellular rejection (ACR) and antibody-mediated rejection (AMR). ACR is mainly mediated by T cells and is the most common type of rejection after cardiac transplantation, usually occurring within 3 to 6 months after transplantation. The inflammatory manifestations include T cell infiltration and cytokine secretion like IL-2, TNF-α, leading to myocardial injury and necrosis (251). AMR is caused by B cells producing antibodies against donor antigens, leading to graft injury through complement activation and inflammatory cell infiltration (252). Chronic rejection, also known as cardiac allograft vasculopathy (CAV), is one of the long-term complications after cardiac transplantation. The inflammatory manifestations include intimal thickening of the coronary arteries, leading to luminal stenosis (253).

The accumulation of macrophages and rejection macrophages has long been considered a characteristic of allograft rejection, with the total number of graft-infiltrating macrophages being associated with poorer clinical outcomes (28, 254). The heart contains both CCR2^+^ and CCR2^−^ macrophages, which exhibit different phenotypes under homeostatic conditions (17). After transplantation, the macrophage population in the heart includes donor-derived CCR2^+^ and CCR2^−^ macrophages, as well as recipient-derived neutrophils, monocytes, monocyte-derived macrophages, and monocyte-derived dendritic cells (255–257). These cells play different roles in rejection and tolerance. CCR2^+^ macrophages are enriched in proinflammatory genes, and their activation represents a mechanism that drives inflammation; CCR2^−^ macrophages promote coronary arteriogenesis and cardiomyocyte proliferation through the secretion of IL-10 and TGF-β and possess potential anti-inflammatory effects (17). Studies have found that MEK1/2 promotes the pro-inflammatory phenotype and glycolytic capacity of alloimmune infiltrating macrophages (AIMs) by regulating PKM2 expression and nuclear translocation, playing a key role in cardiac transplant rejection (258). Further research is needed to elucidate the roles of macrophage metabolism and polarization in cardiac transplant rejection, including their mechanisms in various rejection types and potential therapeutic targets.

#### 3.2.5 Valvular heart disease

Valvular heart disease can be categorized into primary and secondary types. Primary (or degenerative) disease is caused by intrinsic dysfunction of the valve itself, whereas secondary (or functional) disease results from underlying myocardial pathology (259). Inflammatory responses in valvular heart disease involve the activation of endothelial cells and immune cells, which secrete inflammatory cytokines such as TGF-β1, TNF-α, IL-1β, and IL-2, and they promote disease progression (260).

Valvular heart disease is a leading cause of disability and diminished quality of life and is a major contributor to cardiovascular morbidity and mortality worldwide (261). In recent years, an increasing number of studies have highlighted the significant role of macrophages in the development of valvular heart disease (262). In myxomatous valve disease (MVD), there is an increase in the number of CCR2^+^ monocytes and CD206^+^ macrophages, and the accumulation of these cells in immune-active extracellular matrix remodeling areas of the valve leaflets may be associated with disease progression (263). In calcific aortic valve disease (CAVD), macrophages increase infiltration and maturation in response to inflammatory factors secreted by aortic valve cells, thereby promoting osteogenic calcification, affecting the splicing of STAT3 and reducing the expression of the STAT3β isoform, which may promote disease progression (264). Macrophages, particularly the M2 subtype, play a key role in calcific aortic stenosis, and their expression of the pattern recognition receptor Toll-like receptor 7 (TLR7) is associated with inflammation and calcification processes in the aortic valve (265). The activation of TLR7 leads to increased secretion of the immunomodulatory cytokine IL-10, which may help mitigate inflammatory responses and promote valve tissue repair (265). Few studies have explored the role of macrophage metabolism and polarization in valvular heart disease. More research should be devoted to understanding their specific mechanisms in disease progression and repair.

#### 3.2.6 Heart failure

Heart failure (HF) is a multifactorial cardiovascular disease characterized by impaired cardiac pumping function, leading to inadequate organ perfusion and oxygenation (266). Classified by left ventricular ejection fraction (LVEF), HF is typically categorized into heart failure with reduced ejection fraction (HFrEF), heart failure with mildly reduced ejection fraction (HFmrEF), and heart failure with preserved ejection fraction (HFpEF) (266). Endothelial inflammation, activation of the innate immune system, and humoral immunity can all affect the structure and function of the heart, leading to heart failure (267). Heart failure itself can also cause sterile inflammation, with cardiomyocytes and cardiac fibroblasts releasing pro-inflammatory cytokines (such as TNF-α, IL-6, IL-1β) when subjected to mechanical stretching and increased wall tension, activating the NLRP3 inflammasome and further exacerbating inflammation (268). Acute myocardial infarction (AMI) is a major cause of heart failure (269). When AMI occurs, Ly6C^high^ monocytes are the first monocytic population to arrive. They differentiate into M1 macrophages, produce proteases, and secrete MMPs that degrade dying cardiomyocytes and the extracellular matrix (ECM) (270). Hypoxia-inducible factors (HIFs) rapidly accumulate in the nuclei of macrophages in response to the hypoxic microenvironment (271). HIFs promote the transcription of the M1 macrophage gene profile and shift glycolysis to become the primary mode of metabolic energy production in M1 macrophages by driving the expression of the glycolytic gene Slc2al (272).

Clinically, in HF patients undergoing left ventricular assist device implantation, the proliferation of CCR2^+^ macrophages is correlated with the deterioration of left ventricular function and chamber dilation (255). Moreover, these CCR2^+^ macrophages enriched in the hearts of these patients also produce large amounts of IL-1β upon exposure to necrotic cardiomyocytes. IL-1β is known to predict adverse outcomes in HFrEF patients (273). In myocardial biopsies from HFpEF patients, the number of cardiac macrophages is doubled (274), and there is an increase in the gene expression of profibrotic TGF-β (275). These events seem to contribute to the activation of fibroblasts and excessive deposition of collagen (274, 275). Cardiac macrophages also secrete galectin-3, which promotes myocardial fibrosis by activating myofibroblasts (276), as well as phagocytosis-induced TGF-β expression (277). In HFpEF patients, plasma levels of galectin-3 are significantly increased, and elevated levels are associated with a worse prognosis (278).

#### 3.2.7 Arrhythmias

Arrhythmias refer to abnormalities or perturbations in the normal activation or beating of the myocardium. The normal cardiac rhythm is initiated by the sinoatrial (SA) node and conducted through the atrioventricular (AV) node and the His-Purkinje system, resulting in systematic ventricular depolarization. There are numerous types of cardiac arrhythmias. Sinus rhythm, the normal cardiac rhythm, can be disrupted by sinoatrial node dysfunction or inappropriate sinus tachycardia. Additionally, premature atrial contractions and premature ventricular contractions may occur. The severity of arrhythmias is associated with the presence or absence of structural heart disease. Atrial fibrillation (AF) is generally considered benign; however, structural heart diseases such as coronary artery disease or left ventricular dysfunction superimposed with AF may lead to heart failure or sudden cardiac death (279). Inflammation induces atrial arrhythmias by promoting atrial fibrosis, abnormal regulation of gap junctions, and intracellular calcium regulation, causing atrial electrical and structural remodeling (280). Inflammatory markers such as CRP, hs-CRP, and IL-6 are associated with the incidence and duration of atrial fibrillation (280). Following myocardial infarction, macrophages shift toward the M1 phenotype, which impacts the electrical properties of cardiomyocytes via gap junctions and KCa3.1 channel. This leads to variability in cardiomyocyte action potential duration (APD) and contributes to the development of arrhythmias (281).

In both human and murine atrioventricular nodes, CCR2^−^ and CCR2^+^ macrophages are present (282). Cardiac macrophages facilitate signal transmission between cardiomyocytes through connexin 43, thereby maintaining cardiac impulse conduction. Macrophage-produced dual-regulatory proteins are key mediators controlling the phosphorylation and translocation of connexin 43 (206, 283). *In vivo*, the depletion of cardiac resident macrophages or macrophage gap junction protein 43 leads to impaired cardiac conduction (282). Macrophages can promote sympathoexcitation, and proinflammatory factors produced by macrophages, such as IL-1β, IL-6, and TNF-α, can activate the sympathetic nervous system through the circulation or afferent fibers, forming a substrate for proarrhythmia and directly affecting cardiac electrophysiology (284). Recent studies have shown that in atrial fibrillation, the expansion of a subset of macrophages, particularly those positive for secreted phosphoprotein 1 (SPP1), plays a key role in the development of arrhythmias; they promote inflammation and fibrosis through interactions with local cardiac immune and stromal cells, providing a conducive environment for the occurrence and maintenance of atrial fibrillation (285). Currently, there are limited studies on the role of macrophage polarization and metabolic changes in arrhythmias, and more researches are needed to fill this gap.

#### 3.2.8 Aortic aneurysm

Aortic aneurysm (AA) is a chronic aortic disease characterized by permanent, localized dilatation of the aorta resulting from pathological remodeling of the aortic wall. It can further progress to fatal aortic rupture, which is associated with a high mortality rate (286). Aortic rupture is not only associated with the continuous enlargement of the aneurysm diameter; multiple pathological processes also contribute to this progression, including ECM degradation, inflammation, phenotypic transformation of vascular smooth muscle cells (SMCs), oxidative stress, and neovascularization (287). Macrophages play a crucial role in all stages of aortic aneurysm development. M1-type macrophages promote the progression of AA by secreting inflammatory factors and MMPs, which facilitate extracellular matrix destruction and apoptosis of vascular smooth muscle cells (VSMCs). In contrast, M2-type macrophages are mainly involved in vascular repair by inhibiting inflammation (288, 289).

When arteries are damaged, monocytes are recruited to the injury site under the guidance of chemokines such as CCR2 and CCR1, and further differentiate into macrophages (290). Inflammation is one of the core characteristics of aortic aneurysms, and macrophages play a key regulatory role in this process (197). During the clearance of early cellular debris, M1 macrophages generate large amounts of ROS. These ROS act synergistically with ROS derived from endothelial cells, vascular smooth muscle cells, and other immune cells in the aortic wall to continuously activate macrophages, forming a vicious cycle that amplifies the inflammatory response (197, 291). Additionally, M1-type macrophages can secrete pro-inflammatory cytokines such as TNF-α and IL-1β, which further drive the inflammatory process (289, 292). In contrast to M1 macrophages, M2 macrophages secrete anti-inflammatory factors such as IL-10 and TGF-β to inhibit the production of inflammatory mediators and MMPs, and they can also clear free hemoglobin and regulate oxidative stress, which will promote vascular remodeling and repair (288, 293). This process may involve the upregulation of the transcription factor Krüppel-like factor 6 (KLF6) or the downregulation of PPARδ activity (294, 295).

### 3.3 Sex difference in macrophage polarization and inflammatory heart disease

Sex differences also have an impact on macrophage polarization and inflammatory heart diseases. In female mice infected with CVB3, the infiltrating macrophages in the myocardium predominantly exhibit M2 polarization, which alleviates the severity of myocarditis by modulating the local cytokine environment and promoting the differentiation of regulatory T cells (243). In atherosclerosis models, aged female mice have aortas enriched with pro-inflammatory Il1b^+^ M1-like macrophages, showing a stronger inflammatory phenotype, while aged male mice exhibit increased Trem2^+^ macrophages, including anti-inflammatory foam cells and M2-like macrophages (296). Interestingly, another study showed that in female patients, atherosclerotic plaques contain a relatively higher proportion of anti-inflammatory M2 macrophages, which promote inflammation resolution and plaque stability by phagocytosing apoptotic cells and secreting anti-inflammatory cytokines (297); in contrast, male plaques demonstrate more significant infiltration of pro-inflammatory M1 macrophages and inflammation-associated monocyte subsets, such as intermediate monocytes (298). Further research is needed to elucidate the impact of macrophages in different sex groups on inflammatory heart diseases.

### 3.4 New research hotspots: immunosenescence and trained immunity

#### 3.4.1 Immunosenescence of macrophages in inflammatory heart disease

The immune system is essential for defending the body against external pathogens and maintaining homeostasis. However, as people age, the functionality of the immune system gradually declines, a process known as “immunosenescence.” This phenomenon describes the weakening of the immune system associated with aging, characterized by alterations in immune cell populations and functional impairments of both innate and adaptive immunity. As a result, immunosenescence leads to increased susceptibility to infections and various chronic inflammatory diseases (299). Moreover, the aging of the immune system is a cause of immune dysfunction, which can eliminate senescent cells and lead to acquired tissue damage (300). Inflammaging is a chronic, sterile, low-grade inflammatory state associated with aging and is an important hallmark of immunosenescence. This condition is characterized by persistent immune activation and elevated levels of pro-inflammatory cytokines in the circulation, such as TNF-α, IL-6, and IL-1β (301).

The role of immunosenescence in inflammation-related heart diseases is increasingly being investigated. During the process of inflammaging, macrophages accumulate in the arterial wall, where they take up lipids from the blood, particularly LDL, leading to the formation of foam cells. As foam cells accumulate within the arterial wall, they contribute to the formation of atherosclerotic plaques, resulting in vessel stenosis or occlusion (219). With advancing age, the production of NAD^+^ and the levels of NAD^+^-dependent enzymes, such as the SIRT family, decrease in macrophages. This reduction affects the regulation of NF-κB activity, leading to heightened inflammatory responses (302). In aged individuals, the prolonged course of wound repair is associated with prolonged inflammation, delayed neovascularization, and delayed restoration of the extracellular matrix (303). Studies have shown that in aged mice following myocardial infarction, there is impaired inflammation characterized by decreased and delayed neutrophil and macrophage infiltration, reduced cytokine and chemokine expression, and impaired phagocytosis of dead cardiomyocytes. These factors may lead to maladaptive vascular remodeling and tissue repair in the healing heart, accelerating the transition to heart failure (304).

#### 3.4.2 Trained immunity of macrophages in inflammatory heart disease

Previous researchers believed that innate immune cells, such as macrophages, could only non-specifically eliminate pathogens through biological processes like phagocytosis. However, an increasing number of studies have shown that monocytes/macrophages may also develop memory capabilities similar to those of the adaptive immune system after exposure to pathogens (305). Myeloid cells of the innate immune system become more sensitive after activation with the same or different stimuli, resulting in a persistent inflammatory monocyte/macrophage phenotype. This phenomenon is known as “trained immunity” or “innate immune memory” (306). This persistent overactivation of the innate immune system may promote the progression of inflammatory cardiovascular diseases. Risk factors for cardiovascular diseases, such as diet, smoking, hypercholesterolemia, and diabetes, are often associated with low-grade inflammation, which can induce trained immunity and subsequently affect bone marrow hematopoietic progenitor cells through epigenetic modifications, leading to chronic metabolic disorders and vascular inflammation (307). Bekkering et al. (308) proposed that trained innate immunity is associated with the progression of atherosclerosis. Wang et al. (309) also found that trained monocytes/macrophages undergo metabolic and epigenetic changes that keep these cells in a state of chronic overactivation, thereby exacerbating atherosclerosis. Myocardial infarction reprograms bone marrow monocytes through epigenetic modifications, and transplantation of bone marrow from infarcted mice to other mice results in aggravated atherosclerosis and increased infiltration of myeloid cells into plaques (310).

## 4 Macrophage-based target therapy in inflammatory heart disease

As research on macrophages in heart diseases deepens, an increasing number of studies have focused on regulating macrophage polarization, inflammation, and metabolism as potential therapeutic approaches for inflammatory heart diseases (Table 4).

**Table 4:** Summary of macrophage-based target therapy in inflammatory heart disease.

**Disease**	**Therapeutic target/intervention**	**Mechanism of action**	**References**
Atherosclerosis	Clo-Lip	Deplete macrophages via programmed cell death; improve systolic flow velocity	(312, 313)
	Antihyperglycemics (metformin, pioglitazone, sitagliptin)	Induce macrophage M2 polarization; exert anti-atherosclerotic effects	(314, 315)
	Natural compounds (curcumin, polyphenols)	Induce macrophage polarization and regulate macrophage function	(316, 317)
	Novel drug delivery systems (nanoparticles, etc.)	Target macrophage markers (CD11b/163/206); enhance drug bioavailability/specificity	(318)
	MicroRNAs (miR-33/anti-miR-33)	miR-33: Inhibit ABCA1, reduce cholesterol efflux Anti-miR-33: Promote M2 polarization	(319, 320)
Myocardial infarction	NPM1(knockout, ASO-NPM1, NSC348884)	Inhibit mTOR; enhance mitochondrial OXPHOS; induce reparative macrophages; alleviate injury/remodeling	(321)
	TREM2 (overexpression, 4-OI)	Enhance macrophage anti-inflammation; improve cardiac function/remodeling	(233)
	Dimethyl fumarate	Increase OXPHOS; inhibit HIF-1α; promote M2 polarization; improve repair	(322)
Other inflammatory heart diseases	IL-6 (statins, IL-6 inhibitors)	Statins: Reduce IL-6, improve HFpEF prognosis Inhibitors: Improve HFpEF clinical status	(323–325)
	FAP (FAP-targeted CAR-Ms)	Alleviate ischemia-reperfusion fibrosis; improve cardiac function	(326)
	Depletion of CCR2+ macrophages	Inhibit mitral valve disease progression	(327)

### 4.1 Treatment for atherosclerosis

In atherosclerosis, the infiltration of macrophages is closely related to plaque growth and instability. Therefore, depleting macrophages has emerged as a potential therapeutic strategy (311). Using clodronate liposomes (Clo-Lip) to deplete macrophages through inducing programmed cell death has been shown to improve systolic flow velocity in patients with atherosclerosis (312, 313). Some antihyperglycemic drugs, such as metformin, pioglitazone, and sitagliptin, have been found to induce macrophages to shift to the M2 phenotype (314, 315). These drugs exert their anti-atherosclerotic effects by influencing the phenotype of macrophages. Natural pharmaceutical compounds, like curcumin and polyphenols, can also induce macrophage polarization (316, 317). New drug delivery systems, including nanoparticles, stents, oligopeptide complexes, liposomes, and monoclonal antibodies, can selectively modify macrophages. Targeting macrophage surface markers (CD11b, CD163, CD206) in atherosclerotic plaques *in vivo* can achieve macrophage-targeted drug delivery, significantly enhancing drug bioavailability and specificity (318). In lipid metabolism, many microRNAs regulate cholesterol efflux by targeting ATP-binding cassette transporter (ABCA1/ABCG1). For example, miR-33 inhibits ABCA1 expression, it can reduce cholesterol efflux (319). Anti-miR-33 nano therapy can modulate the polarization of macrophages to the M2 phenotype. Targeting microRNAs in macrophages may be a promising therapeutic approach for atherosclerosis (320).

### 4.2 Treatment for myocardial infraction

Nucleophosmin1 (NPM1) knockout inhibits glycolysis in macrophages after myocardial infarction (MI) by suppressing mTOR signal transduction, enhances OXPHOS, transforms cardiac macrophages into a reparative phenotype, and alleviates myocardial ischemic injury and adverse ventricular remodeling (321). The application of antisense oligonucleotide drugs (ASO-NPM1) and polymerization inhibitors (NSC348884) targeting NPM1 can also significantly promote the transformation of reparative macrophages, thereby improving cardiac function and mitigating ventricular remodeling after MI (321). The protective role of TREM2^+^ macrophages in myocardial infarction has been discussed. Adenoviral-induced TREM2 overexpression and the exogenous administration of the itaconate derivative 4-octyl itaconate (4-OI) can enhance the anti-inflammatory function of macrophages and improve cardiac function and ventricular remodeling after MI (233). Dimethyl fumarate can promote the polarization of macrophages to the M2 phenotype by increasing mitochondrial OXPHOS levels and inhibiting the expression of HIF-1α, improving damage repair after MI (322).

### 4.3 Treatment for other inflammatory heart diseases

IL-6 has the capacity to regulate cardiomyocytes and macrophages and plays a crucial role in the pathogenesis of heart failure with HFpEF (323). Statins can reduce the expression levels of IL-6, contributing to the improved prognosis of patients with HFpEF (324). Inhibition of IL-6 in patients with HFpEF can effectively improve their clinical condition (325). Newly discovered FAP-targeted CAR-Ms can effectively alleviate myocardial fibrosis following myocardial ischemia-reperfusion injury, improve cardiac function, and demonstrate long-term cardioprotective effects (326). In Marfan syndrome (MFS) mice, depletion of CCR2^+^ macrophages can protect against the progression of myxomatous valve degeneration (327). Further investigation is required to develop more therapeutic approaches targeting macrophages.

### 4.4 Limitation of the treatment

The spatial structure of biomolecules (such as cytokines and chemokines) is affected *in vivo* by biological, physical, and chemical factors, including biological enzymes, temperature, and pH value. Additionally, these biomolecules face issues such as off-target effects and difficulty in crossing biological membrane barriers, which to a certain extent hinder the exertion of drug efficacy (328, 329). Nano delivery technologies can reduce off-target effects, help drugs cross barriers, and improve bioavailability; however, the problem of high manufacturing costs urgently needs to be addressed (197). Furthermore, it is difficult to determine the therapeutic time window for different diseases. For example, early inhibition of IL-1β secreted by macrophages in acute myocardial infarction leads to insufficient scar formation and cardiac rupture (330), whereas early inhibition of IL-1β in ischemia-reperfusion injury can reduce infarct size and improve ventricular remodeling (331). In the future, we need more research to solve these problems in practical applications.

## 5 Conclusions and perspectives

The occurrence, development, and prognosis of heart diseases are closely related to immune inflammation. Immune inflammation plays a significant role in the occurrence and development of various heart diseases, and anti-inflammatory therapy has become a research hotspot in the treatment of them. Identifying more anti-inflammatory treatment targets and drugs is highly important. Macrophages, as key immune cells, play a central role during inflammatory heart diseases. A more in-depth investigation into therapies targeting macrophages may bring new hope for inflammatory heart diseases.
